# Direct Solution of the Chemical Master Equation Using Quantized Tensor Trains

**DOI:** 10.1371/journal.pcbi.1003359

**Published:** 2014-03-13

**Authors:** Vladimir Kazeev, Mustafa Khammash, Michael Nip, Christoph Schwab

**Affiliations:** 1 Seminar für Angewandte Mathematik, ETH Zürich, Zürich, Switzerland; 2 Department of Biosystems Science and Engineering, ETH Zürich, Basel, Switzerland; 3 Department of Mechanical Engineering, UC Santa Barbara, Santa Barbara, California, United States of America; 4 Seminar für Angewandte Mathematik, ETH Zürich, Zürich, Switzerland; University of Michigan, United States of America

## Abstract

The Chemical Master Equation (CME) is a cornerstone of stochastic analysis and simulation of models of biochemical reaction networks. Yet direct solutions of the CME have remained elusive. Although several approaches overcome the infinite dimensional nature of the CME through projections or other means, a common feature of proposed approaches is their susceptibility to the curse of dimensionality, i.e. the exponential growth in memory and computational requirements in the number of problem dimensions. We present a novel approach that has the potential to “lift” this curse of dimensionality. The approach is based on the use of the recently proposed Quantized Tensor Train (QTT) formatted numerical linear algebra for the low parametric, numerical representation of tensors. The QTT decomposition admits both, algorithms for basic tensor arithmetics with complexity scaling linearly in the dimension (number of species) and sub-linearly in the mode size (maximum copy number), and a numerical tensor rounding procedure which is stable and quasi-optimal. We show how the CME can be represented in QTT format, then use the exponentially-converging 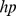-discontinuous Galerkin discretization in time to reduce the CME evolution problem to a set of QTT-structured linear equations to be solved at each time step using an algorithm based on Density Matrix Renormalization Group (DMRG) methods from quantum chemistry. Our method automatically adapts the “basis” of the solution at every time step guaranteeing that it is large enough to capture the dynamics of interest but no larger than necessary, as this would increase the computational complexity. Our approach is demonstrated by applying it to three different examples from systems biology: independent birth-death process, an example of enzymatic futile cycle, and a stochastic switch model. The numerical results on these examples demonstrate that the proposed QTT method achieves dramatic speedups and several orders of magnitude storage savings over direct approaches.

## Author Summary

Stochastic models of chemical networks are necessary to quantitatively describe random fluctuations and other probabilistic phenomena within living cells. The Chemical Master Equation (CME) describes the time evolution of molecular abundance probabilities in these models, and is a basis for many stochastic simulation and analysis methods. Yet the CME is difficult to solve directly except for very simple structures. Indeed current approaches are susceptible to the curse of dimensionality, that is, the exponential growth of memory and computational requirements in the number of problem dimensions. In this paper, we propose a novel approach that has the potential to overcome the curse of dimensionality. It is based on the use of the recently proposed Quantized Tensor Train (QTT) formatted numerical linear algebra for numerical representation of tensors, using algorithms for basic tensor arithmetics with complexity scaling linearly in the number of reacting species considered, and sub-linearly in the maximum allowed copy number per species. We present this approach and demonstrate its effectiveness by applying it to three problems from systems biology. Numerical experiments are reported which show that several orders of magnitude memory savings is typically afforded by the new approach presented here.

This is a *PLOS Computational Biology* Methods article.

## Introduction

In spite of the success of continuous-variable deterministic models in describing many biological phenomena, discrete stochastic models are often necessary to describe biological phenomena inside living cells where random motion of reacting species introduces randomness in both the order and timing of biochemical reactions. Such random effects become more pronounced when one factors in the discrete nature of reactants and the fact that they are often found in low copy numbers inside the cell. Manifestations of randomness vary from copy-number fluctuations among genetically identical cells [1] to dramatically different cell fate decisions [2] leading to phenotypic differentiation within a clonal population. Characterizing and quantifying the effect of stochasticity and its role in the function of cells is a central problem in molecular systems biology.

In order to effectively capture this experimentally observed stochasticity, the evolution of the chemical species of interest are commonly modeled using jump Markov processes. Here, each state of the process corresponds to the copy number of one of the constituent species [3]. Within this framework, the evolution of the probability density over the possible configurations of the reaction network is described by a Forward Kolmogorov Equation, frequently referred to as the Chemical Master Equation (CME) within the chemical literature. While analytical solutions can be obtained under specific assumptions about the structure of the chemical network [4], these assumptions prove so restrictive as to exclude the vast majority of biologically relevant systems. In most cases, the CME cannot be solved explicitly and various numerical simulation techniques have been proposed to approximately solve the time-evolution problem.

A first class of methods seeks to compute approximations of the CME solution instead by solving a truncated version of the original Markov process. These methods are advantageous in that they provide explicit error guarantees after simulation. This class includes the finite state projection [5] and sliding window abstraction [6]. In these methods, the truncation is chosen so that both the number of states retained is small enough that it may be computed efficiently but large enough that it retains the majority of the probability mass over the time evolution. Clearly, these two objectives are not complementary. In order to guarantee that the approximation has low error, most biologically relevant reaction networks require truncations with so many states that they are completely intractable on available hardware. The finite buffer method [7], [8] suggests a more sophisticated truncation to the states reachable from a given initial state assuming that only a prespecified finite number of molecules may be spontaneously created. However, its use is limited to explicit time-stepping schemes, in addition to requiring that the finite buffers be large to compute accurate solutions.

A second broad class of methods are the kinetic Monte Carlo approaches which instead seek to produce either exact or approximate realizations of the underlying Markov process [3], [9], [10]. By generating sufficiently many realizations, these methods obtain statistics for events that are biologically important. Unfortunately, in many systems, these important events occur rarely, so that producing enough realizations to estimate these statistics is prohibitive.

A third class of methods use asymptotic approximations to trade accuracy for computational or analytical tractability. This class includes the Moment Closure methods [11], [12], the Linear Noise Approximation (LNA) [13], and Chemical Langevin Equation (CLE) treatments [14], [15]. Each of these methods replaces the discrete description of the population counts with a continuous one and can therefore perform poorly in situations where the discrete dynamics are difficult to capture with continuum models, e.g. when even one of the reacting species exhibits low population count or is constrained to have low population count, for instance, in the presence of conservation laws.

Some of the classes of methods described so far perform well in complementary regimes and recently there has been substantial effort to combine these methods resulting in the so-called hybrid methods. Several methods require a time-scale separation of the dynamics to split the system into fast and slow species and impose a quasi-stationary assumption for the fast reactions. An approximate method which can converge quickly to an accurate approximation of a stationary distribution such as 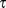-leaping [16] or the Chemical Langevin Equation [17], [18] is used for the fast species, while the slower but more accurate Gillespie algorithm is used for the slow species. Rather than partitioning the species by time-scales of the associated reactions, other methods separate by average molecule count. The low count species are tracked by kinetic Monte Carlo while an ODE approximation is made for the dynamics of the high count species [19], [20]. While these methods allow faster simulations, speedups come at the cost of accuracy, as modeling errors are introduced by the partial replacement of the CME with cruder descriptions.

In order to provide methods that are both accurate and computationally efficient, several numerical techniques for compressing the dynamics and the solution have been explored in the recent literature. Attempts were made to expand the probability distribution as a linear combination of a small set of so-called “principal”, orthogonal basis functions [21]–[25]. Then, either a Galerkin projection was used to map the dynamics onto the lower dimensional subspace spanned by the basis functions (Method of Lines) or first a time discretization was used and then the basis at each time step was adapted by either adding or subtracting basis elements (Rothe's Method). These methods differ primarily in their choice of orthogonal basis. A common feature of these approaches is that they begin with a basis for probability distributions of a single variable and then use the corresponding tensor product basis for multivariate distributions. This means that they are susceptible to the so-called *curse of dimensionality* [26], that is, the memory requirements and computational complexity of basic arithmetics grow exponentially in the number of dimensions. In the context of the CME, this means that all of these approaches can exhibit an exponential scaling of the complexity with the number of chemical species in the model.

Recent papers have attempted to address the curse of dimensionality by using a low-parametric representation of tensors known as *canonical polyadic* decomposition or *CANDECOMP/PARAFAC*, both notions being subsumed under the acronym *CP* [27], [28]. CP is a methodology for generalizing the singular value decomposition (SVD) for matrices to tensors of dimension greater than two by representing the solution as sums of rank-one tensors (equivalently, linear combinations of distributions in which species counts are independent at each fixed time point). As long as the tensor rank of the solution to be approximated remains low, these approaches can be very computationally efficient as basic arithmetics for tensors in the CP format scales linearly in the number of tensor dimensions.

A key challenge in applying the CP decomposition to construct approximate CME solvers is to control the tensor rank of the computed solution. Basic algebraic tensor operations such as addition and matrix-vector multiplication generally increase rank and hence computational cost. In [29] it is suggested to recompute a lower rank CP decomposition after *every* arithmetic operation. This approach turned out to be problematic in practice. One reason is that the problem of tensor approximation (in the Frobenius norm) with a tensor of fixed rank is, in general, ill-posed [30]. Thus, the numerical algorithms for computing an approximate representation may easily fail. Another reason is that the problem is NP-hard [31], [32] and there is no robust algorithm having any affordable complexity.

Another approach [33], related to the present work, attempts to avoid the problem of approximation in the CP format entirely by projecting the dynamics onto a manifold composed of all tensors with a CP decomposition of some predetermined maximal tensor rank. This procedure results in a set of coupled nonlinear differential equations which are then solved using available ODE solvers. While this effectively controls the tensor rank of the approximate solution, still, to the authors' knowledge, there is no way to estimate either theoretically (*a priori*) or numerically (*a posteriori*) the CP rank of the full CME solution as a function of given data.

In this paper we propose a new, deterministic computational methodology for the direct numerical solution of the CME, without modelling or asymptotic simplifications. The approach has complexity that scales favorably in terms of the number of different species considered and the maximum allowable copy number of each of these species. It is based on the recently proposed *Quantized Tensor Train* (QTT) formatted, numerical tensor algebra [34]–[37] which operates on low-parametric, numerical representation of tensors, rather than on their CP representations. This decomposition admits both algorithms for basic tensor arithmetics that scale linearly in the dimension (the species number) and a robust adaptive numerical procedure for the tensor truncation, which is quasi-optimal in the Frobenius norm.

We show in the present paper how the CME can be represented in QTT format, then use 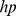-discontinuous Galerkin discretization in time to exploit the time-analyticity of the CME evolution and to reduce the CME evolution problem to a set of QTT structured linear equations that are solved at each time step [38]. We then exploit an algorithm available for solving linear systems in this format that is based on Density Matrix Renormalization Group (DMRG) methods from quantum chemistry.

The numerical experiments reported below (see, in particular, Table 1) show several orders of magnitude memory savings, which is typically afforded by the new approach presented here.

**Table 1:** Overview of the QTT compression of the storage needed for solutions (maximum throughout the time stepping) and CME operators.

	Direct Approach		Proposed Approach					
run	solution	operator	solution		truncated solution		operator	
	Mem	Mem	Mem	ratio	Mem	ratio	Mem	ratio
*d* independent birth-death processes								
*d* = 1	4.10**_3_**	1.68**_7_**	736	1.80_−**1**_	264	6.45_−**2**_	992	5.91_−**5**_
*d* = 2	1.68**_7_**	2.82**_14_**	3858	2.30_−**4**_	528	3.15_−**5**_	2852	1.01_−**11**_
*d* = 3	6.87**_10_**	4.72**_21_**	7742	1.13_−**7**_	898	1.31_−**8**_	4800	1.02_−**18**_
*d* = 4	2.81**_14_**	7.90**_28_**	12176	4.33_−**11**_	1432	5.09_−**12**_	6748	8.52_−**26**_
*d* = 5	1.15**_18_**	1.32**_36_**	16262	1.41_−**14**_	1946	1.69_−**15**_	11032	8.30_−**33**_
genetic toggle switch								
only	3.36**_7_**	1.12**_15_**	65264	1.95_−**3**_	–	–	10988	9.76_−**12**_
enzymatic futile cycle								
(A)	4.19**_6_**	1.76**_13_**	18396	4.39_−**3**_	8472	2.02_−**3**_	25848	1.47_−**9**_
(D)	4.19**_6_**	1.76**_13_**	360332	8.59_−**2**_	290144	6.92_−**2**_	5584	3.17_−**10**_

For details on “truncated solution” see **Numerical experiments. Common details**. Solution 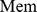 in the Direct Approach is the number of states taken into account in the FSP, which is equal to the number of entries, 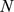, in the solution vector. For the CME operator, 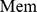 is 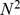, the number of entries in the matrix. In the Proposed QTT Approach, *ratio* indicates the memory storage compression ratio, i.e. the ratio of 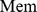 in the Proposed QTT Approach to that in the Direct Approach. In the sparse representation of the CME operator the number of nonzero entries would be 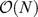 rather than 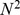. The exponents are given in boldface for the base 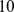.

## Results/Discussion

We start our development by formulating the Chemical Master Equation (CME), arising from stochastically reacting chemical species. Then we will devote the remainder of the article for its proposed solution. A “well-stirred” solution of 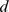 chemically reacting molecules in thermal equilibrium can be described by a jump Markov process, where for each fixed time 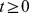, 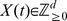 is a random vector of nonnegative integers with each component representing the number of molecules of one chemical species present in the system. In [29] and the references therein, it is shown that, given an initial condition 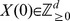, the corresponding probability density function (PDF) 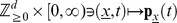 of the process solves the Chemical Master Equation (CME):
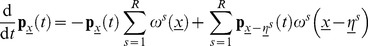
(1)where 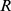 is the number of reactions in the system, 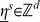 and 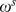 are the stoichiometric vector and propensity function of the 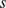
th reaction, respectively. The CME is a system of coupled linear ordinary differential equations with one equation per state 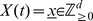.

### Our Approach to Solving the CME

We briefly outline our proposed methodology for the numerical solution of the CME. Since the state space of solutions is countably infinite, the main challenge to be overcome is the curse of dimensionality. As the state space of the CME is typically countably infinite, there is a countably infinite number of different possible states that could be reached by the chemical system. Our approach consists of employing efficient methods for tensor-structured, rank-adaptive numerical solution of very large but “finite state projection” truncations of the CME. In a nutshell, we are proposing to solve large, coupled systems of linear ODEs with a special, tensor structure inherited from the CME. We now give a general outline of our approach, followed by detailed descriptions of each of these steps.

#### 1. Truncate the CME to obtain a linear ODE with a finite state space

The CME describes the dynamics of probabilities of finding the chemical system in different states. In general the number of these different states is countably infinite, as it is not unknown *a priori* the maximum number of copies that each species can take. While this gives rise to an infinite number of state variables, each indicating the probability of a given chemical state, the vast majority of these probabilities are vanishingly small. This has motivated approaches for truncating the infinite number of state variables in the CME in a way that results in a finite number of state variables corresponding to chemical states that are likely to have high probability mass. The truncated CME consists of a system of linear ODEs with finite state space, that can *in principle* be solved. One such truncation approach which we will follow here is the Finite State Projection method. This truncation approach has the advantage of yielding bounds on the error between the solution of the truncated finite system and the original infinite set of ODEs (the CME). The Finite State Projection has been reported elsewhere [5], but we give a brief description of the approach in this article for completeness.

#### 2. Express the truncated CME using tensors; Employ numerical rank reduction and compression to save storage and to speed up algebraic operations

In conventional approaches to solving the CME, the state-space is enumerated by a “long” index and the corresponding probabilities are stacked into a vector 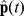 that is then multiplied by the CME operator to form the right-hand side of the ODE: 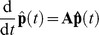. At all times 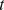 the solution is an array indexed by a multi-index, e.g., 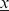, which is a 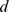-tuple of indices 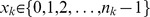, where 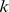 ranges from 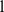 to 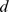. We shall also refer to 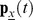 as a 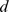-dimensional 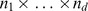-vector. Our approach is based on exploiting the high-dimensional structure of the vectors and matrices involved, related to the separation of the indices, instead of stacking all indices into a single “long” index.

Linear operators acting on these 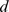-dimensional 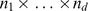-vectors can themselves be expressed using tensor notation. In our case, the action of the CME operator 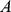 is one such operator. A key aspect of our approach is that both the tensor vector 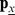 and the tensor operator 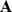 arising from CME problems admit a dramatic level of compression. This tensor compression is achieved through the so called tensor train representation (TT). Tensor train compression goes beyond exploiting the sparsity structure, and actually exploits the rank structure of the tensor. This reduced rank compression is at the heart of our approach to the CME solution. The compression itself is analogous to the compression of the low-rank representation of usual matrices. Indeed, an 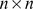 matrix of rank 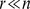 can be stored using 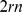 variables, while the approximation can be based, e.g., on the Singular Value Decomposition (SVD). In a similar fashion, the TT format is a generalization of this compression to multidimensional tensors. This is both true for tensors and linear operators acting on these. Further *adaptive data reduction and compression* is afforded by the so-called quantized tensor train (QTT) format. Both the TT and QTT formats will be discussed later in this article, along with simple examples demonstrating the compression that can be achieved by using these formats.

#### 3. Employ 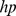-discontinuous Galerkin discretization in time to solve the truncated ODE

Once the problem has been represented in QTT format, we use 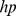-discontinous Galerkin (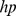-DG) discretization in time as the time-stepping scheme [38] to solve the truncated ODE. Given a time mesh, the 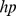-DG method finds an approximate solution to the initial value problem that is a polynomial when restricted to each subinterval of the time mesh and possibly discontinuous at each mesh point. This method allows adaptation of the size of each time step (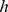-adaptation), allowing good resolution of fast transients, as well as the order of the approximating polynomial on each time step (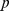-adaptation), or both simultaneously (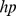-adaptation). For linear ODE initial value problems like the projected CME, the 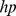-DG approach achieves *exponential rates of convergence* to the classical solution with respect to the number of temporal degrees of freedom. Practically, 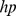-DG discretization in time reduces the projected CME evolution problem to a sequence of systems of QTT structured linear equations that must be solved at each time step. Our computational method then exploits an algorithm available for solving linear systems in this format that is based on Density Matrix Renormalization Group (DMRG) methods from quantum chemistry.

### CME Truncation: Separability and Finite State Projection of the CME Operator

Munsky and Khammash [5] rewrote the right-hand side of the CME (1) as the action of a linear operator 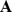 on the probability density at the current time:
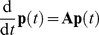
(2)Throughout this paper, we refer to 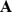 as the *CME operator*.

Hegland and Garcke introduced an explicit representation of the CME operator as sums and compositions of a few elementary linear operators [29]: let 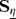 be the spatial shift of a probability density by a vector 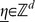 and let 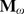 be multiplication by a real-valued function 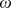:
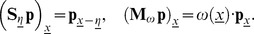
Then the CME operator can be written as follows, with 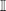 denoting the identity operator:
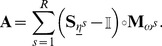
(3)To simplify the exposition, we assume that all propensity functions are *rank-one separable*, i.e. they are of the form
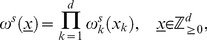
(4)for 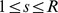, where each 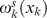 is a nonnegative function in the single variable 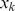. Considering rank-one separable propensity functions is sufficient for all elementary reactions which occur as building blocks in more complicated reaction kinetics.

The CME (2) is posed on the (countably) infinite space 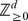 of states. In this form, the CME (1) is an infinite-dimensional coupled evolution problem which necessitates truncation prior to numerical discretization. In the case of a particular class of monomolecular reactions, Jahnke and Huisinga were able to construct an explicit solution in terms of convolutions of products of Poisson and multinomial distributions [4].

In order to be able to address more complex systems computationally, Munsky and Khammash proposed the Finite State Projection Algorithm (FSP) [5] which seeks to truncate the countably infinite dimensional space 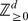 of states of the process to its finite subset
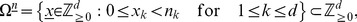
(5)associated with a multi-index 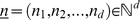, so that the dynamics over 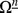 are close to those of the original system; see Theorem 1. In practice, the truncation satisfying a given error tolerance may still require a very large number of states: the dimension of the FSP vector 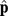 equals 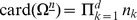 rendering a direct numerical solution of even the projected equation (S1.1) infeasible in many cases. The remainder of the paper presents a novel approach for the numerical solution of such FSP truncated systems that retain large numbers of states. For notational convenience, we drop the superscripts 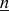 and the hat from 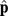 indicating the FSP since we will only consider systems which have already been truncated. Similarly, we now use the shift and multiplication operators in (3) restricted to the truncated state space without change of notation.

Assuming that a FSP has been performed, we henceforth treat 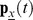 as a 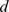-dimensional 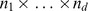-vector, i.e. as an array indexed by 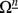 which we identify with *ordered* 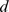-tuples of indices 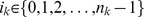, where 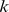 ranges from 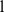 to 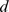. Each dimension 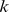 (alternatively referred to as a *mode* or *level*) has a corresponding *mode size* 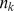, that is, the number of values which the index for that dimension can take. For our chemically reacting system, 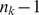 corresponds to the maximum number of copies of the 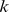
th species that is considered. For a more detailed introduction to basic tensor operations and terminology see, for example, [39], [40].

For the same ordering of 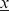, consider the corresponding *d*-dimensional 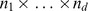-vectors 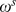, 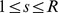, containing the values of the propensities on 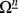 to which we shall refer as *propensity vectors*:
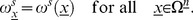
(6)Within the projected CME (S1.1), the operators corresponding to weighting by the propensity functions, involved in (3), are finite matrices: 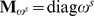. Then, under the rank one separability assumption (4), with 
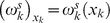
 for 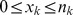, 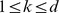 there holds
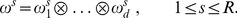
(7)


### Tensor Representation of the CME: TT and QTT Formats

#### Tensor Train representation of vectors and matrices

**Figure 1:**
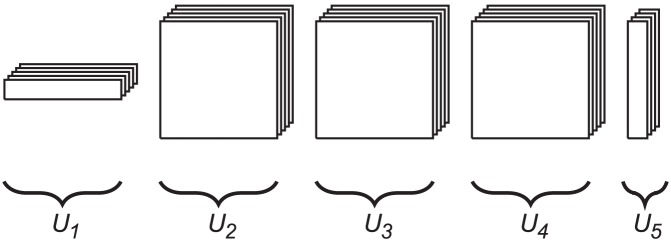
Schematic drawing of a TT decomposition of a five-dimensional array. Each TT core can be visualized as a stack of matrices with the size of the stack equal to the corresponding mode size. The number of TT cores is equal to the number of dimensions of the array. Element 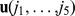 of the full array is given by the (matrix) product of matrix 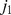 selected from core 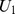, matrix 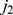 from core 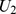, etc. Note that the size of each matrix within a core must be the same, but may differ between distinct cores. Note also that the number of matrices in each core depends on the corresponding mode size of the full tensor and generally differs between cores. Such an interpretation in the sense of a product of parametric matrices is widely used for the *Matrix Product States*, see [46]–[48].

Our approach to the direct numerical solution of the CME (2) is based on the structured, low-parametric representation of all vectors and matrices involved in the solution in the *Tensor Train* (TT) format [34], [41] developed by Oseledets and Tyrtyshnikov. To present it, let us consider a 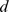-dimensional 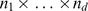-vector 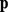 and assume that two- and three-dimensional arrays 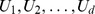 satisfy
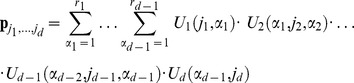
(8)for 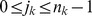, where 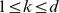. Then 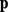 is said to be represented in the TT decomposition in terms of the *core tensors* 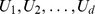. The summation indices 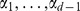 and limits 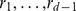 on the right-hand side of (8) are called, respectively, *rank indices* and *ranks* of the decomposition. See Figure 1 for a schematic drawing.

The TT decomposition can potentially result in large compression of the tensor by exploiting the rank structure of the tensor. This is demonstrated in a simple example

**Example 1 (TT Compression)** *Consider a vector* 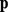 *of size* 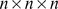 *given elementwise by*

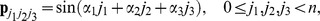

*where* 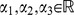 *. By applying trigonometric identities, one obtains for all* 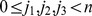 *the following row-matrix-column factorization:*

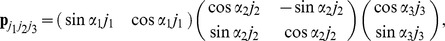

*in which the indices are separated so that every factor depends on the corresponding index only. This factorization implies a TT representation of the form (8) with ranks* 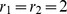 *and the cores given for* 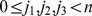 *by*

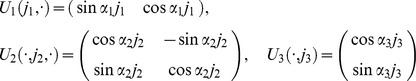

*This TT decomposition involves* 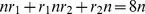 *parameters instead of* 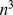 *required for the elementwise representation. The case of* 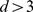 *dimensions is considered in* [*42, Theorem 4*] *, the number of parameters being under* 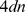 *compared to* 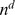.

Unlike CP, the TT format allows the construction of a decomposition, exact or *approximate*, through the low-rank representation of a sequence of single matrices; for example, by SVD. In particular, note that for every 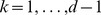 the decomposition (8) implies a rank-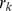 representation of an *unfolding matrix* 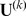 which consists of the entries
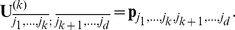
Here, the overscore denotes the vectorization of multi-indices: 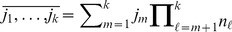 for 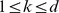. Conversely, if the vector 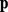 is such that the unfolding matrices 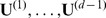 are of ranks 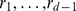 respectively, then the cores 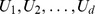, such that (8) holds, do exist; see Theorem 2.1 in [41]. The ranks of the unfolding matrices are the lowest possible ranks of a TT decomposition of the vector and, therefore, are called *TT ranks of the vector*.

What is more important is that the low-rank matrix structure of the unfolding matrices translates into the low-rank TT structure of the vector. Once the former can be approximated in the Frobenius norm with ranks 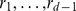 and accuracies 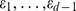, the latter can be approximated in the same norm in the TT format with ranks 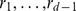 and accuracy 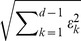. The proof relies on the TT approximation algorithm. For details, we refer to Theorem 2.2 with the corollaries and to Algorithms 1 and 2 in [41]. This low rank approximation of the unfolding matrices can be considered and is implemented as adaptive and compressive data representation at each stage of computation.

**Algorithm 1.** Assemble Projected CME Operator in QTT Matrix Format.

**Require:** Rank-1 separable propensity functions 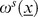, stoichiometric vectors 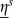, rectangular FSP truncation 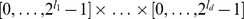, propensity QTT compression tolerance 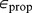, a QTT approximation subroutine 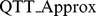 implementing [4, Algorithm 1] for quantized vectors.

**Ensure:** Projected CME operator 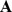 in QTT matrix format

Initialize 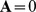;
**for** 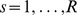 **do**
  

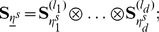

  
**for** 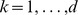 **do**
   

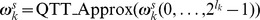
 with tolerance 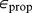;
  
**end for**
  

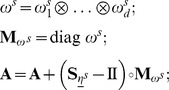

  
**end for**

**Algorithm 2.** 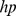-DG-QTT CME Solver.

**Require:** Projected CME operator 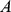 in QTT format, time mesh 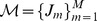, polynomial orders 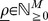, basis of temporal shape functions 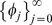, DMRG-solver tolerance 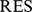

**Ensure:** Approximate solution 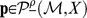 of the evolution 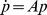

**for** 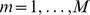 **do**
  
**for** 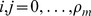 **do**
   

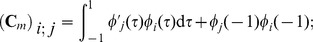

   

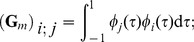

  
**end for**
  Solve 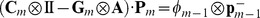, for 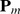

  using DMRG-solver with tolerance 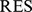;
  

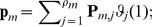

**end for**

**Example 2 (Unfolding of a tensor)** *Consider a tensor* 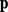 *of size* 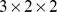 *. It has two unfolding matrices* 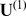 *and* 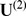 *given by*

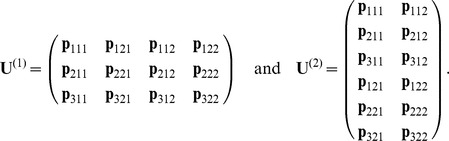

*While* 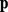 *,* 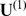 *, and* 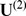 *are structured differently, all have the same entries and represent the same data. The two TT ranks of* 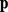 *are exactly the (matrix) ranks of* 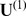 *and* 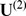.

Note also that, unlike CP, the TT representation relies on a certain ordering of the dimensions so that *reordering dimensions may affect the numerical values of TT ranks significantly*. We discuss this issue in more detail in the transposed QTT section.

The TT representation may be applied to multidimensional matrices in a similar way as to vectors. Consider a 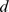-dimensional 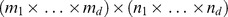-matrix 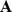. Let us vectorize it and merge the corresponding row and column indices to obtain a 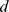-dimensional 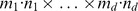-vector 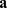. Then the TT representation of the vector 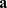, given by the elementwise equality
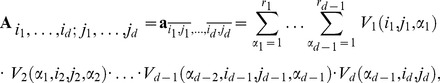
(9)is called a TT representation of the matrix 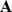, the cores 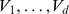 are now three- and four-dimensional arrays. Our discussion of the efficiency and robustness of the TT decomposition of vectors also applies to the matrix case.

Note that the *Hierarchical Tensor Representation* [43], [44] itself and coupled with the *tensorization* [45], an extensive overview of which is available in [40], are closely related counterparts of the TT and QTT formats respectively. Also, the structure called now TT decomposition has been known in theoretical chemistry as *Matrix Product States (MPS)*. It has been exploited by physicists to describe quantum spin systems theoretically and numerically for at least two decades now, see [46], [47], cf. [48].

Basic operations of the numerical calculus with vectors and matrices in the TT format, such as addition, Hadamard and dot products, multi-dimensional contraction, matrix-vector multiplication, etc. are considered in detail in [41]. Since the main aim of using tensor-structured approximations is to reduce the complexity of computations and avoid the curse of dimensionality, we emphasize that the storage cost and complexity of basic operations of the TT arithmetics, applied to the representation (8), can be bounded by 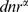 with 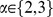, where 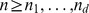 and 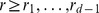. This estimate is formally linear in 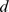; however, the TT ranks 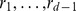 in (8) may depend on 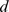 and 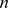. Showing that the TT ranks are moderate, e. g. constant or growing linearly with respect to 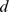 and constant or growing logarithmically with respect to 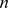, is a crucial issue in the context of TT-structured methods and has been addressed so far mostly experimentally, see, e. g. [49]–[53].

The TT approximation of a vector with 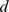 indices treated separately results in a decomposition with 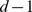 TT ranks which may take different values. To characterize them, an aggregate characteristic such as the *effective rank* of the TT decomposition is used. Consider an 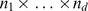-tensor is given in a TT decomposition with ranks 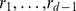. We call the positive root 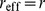 of the quadratic equation
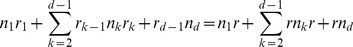
(10)the *effective rank of the decomposition*. Note that, for integer values of 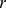, the definition (10) equates the memory needed to store two TT representations. The one corresponding to the left-hand side, is the given decomposition. The one corresponding to the right-hand side is of a vector with the same mode sizes, but with equal 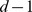 ranks 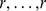. This renders 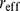 “effective” with respect to memory. On the other hand, the complexity of some TT-structured operations, such as the matrix-vector multiplication and Hadamard product, can also be estimated with the use of 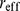.

#### Quantized Tensor Train representation

So far, we have discussed the use of the TT format for extracting low-rank structure with respect to the “physical” dimensions, which are naturally distinguished in the data due to their meaning in the context of a particular problem. For the subject of the present paper, such dimensions represent the reacting species. However, every such a dimension can be unfolded, or *quantized*, into a few virtual dimensions representing its levels, or scales. Then the data can be represented in the TT format applied to all the virtual dimensions introduced. The use of quantization in the context of tensor decompositions dates back to [54]. For the TT format, it results in the *Quantized Tensor Train* (QTT) format [35]–[37]. For the convenience of the reader, we provide a brief review and refer to [35]–[37] for details.

Consider a 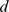-dimensional vector of size 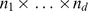. Assume that the 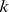
th mode size 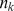 can be factorized as 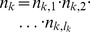 in terms of integral factors 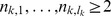. Then the 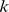
th mode index 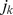 can be represented in a one-to-one fashion through a tuple 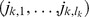 of 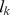 *virtual* indices. Here, 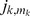 runs from 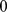 to 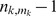 for 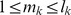. The index transformation rule can be defined in many ways.

In order to associate the virtual indices with the scales in the vector, the transformation 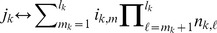 can be chosen. This index (bijective) transformation is analogous to the positional notation for encoding numbers. In this work we indicate this by the overscore notation 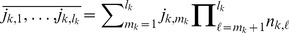. In the most general case, the “virtual” mode factors 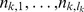, which are analogous to the bases in the positional notation, may differ for different positions 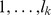.

In terms of the vector, such an index transformation is often called *quantization*. It is equivalent to folding, or reshaping, the 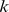
th mode of size 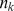 into 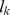 modes of sizes 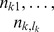. When applied to all dimensions, this procedure transforms a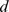-dimensional 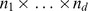-vector indexed by 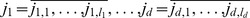 into an 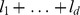-dimensional 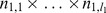 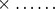 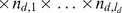-vector indexed by 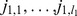, 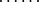, 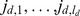. A TT decomposition of the quantized vector is referred to as *QTT decomposition* of the original vector; the ranks of this TT decomposition are called *ranks of the QTT decomposition* of the original vector. For details, we refer to the papers [35]–[37] cited above.

**Example 3** (QTT Compression) *Consider a vector* 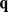 *of size* 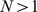 *given elementwise by*

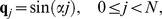

*where* 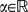 *. Assume that* 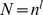 *, where* 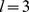 *and* 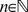 *. Then the index* 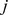 *running from 0 to* 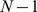 *can be represented as* 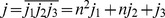 *through* 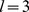 *“virtual” indices* 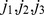 *running from* 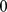 *to* 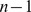 *each. The corresponding quantization* 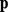 *of* 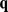 *of size* 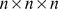 *is given by*

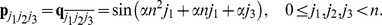

*By applying the discussion of Example 1 to* 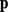 *, we see that the QTT format represents* 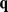 *with the cores and ranks given in Example 1 through* 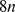 *parameters intead of* 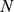 *required for the elementwise representation. The case of* 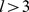 *virtual levels is considered in* [*37, Lemma 2.5*] *and* [*42, Theorem 7*] *, the number of parameters being under* 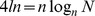 *instead of* 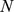 *. In these paper, we use the binary quantization with* 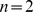.

If the natural ordering
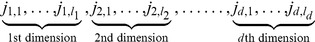
(11)of the “virtual” indices is used for representing the quantized vector in the TT format, then the ranks of the QTT decomposition can be enumerated as follows:
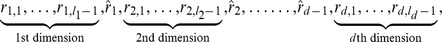
where 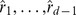 are the TT ranks of the original tensor, i.e. the ranks of the separation of “physical” dimensions. That is, the TT ranks of a tensor are some of the QTT ranks of the same tensor, provided that the natrual ordering (11) is used.

Note that equations (8) and (9) can also be understood as QTT representations of a “one-dimensional” vector (i.e. a vector with a single “physical” index) 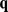 and of a “one-dimensional” matrix (i.e. a matrix transforming such vectors) 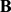 with entries 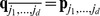 and 
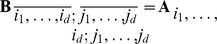
 respectively. In this case, 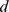 denotes the number of virtual dimensions corresponding to the single “physical” dimension.

As a QTT decomposition is a TT decomposition of an appropriately quantized (and possibly, as we discuss in a later section, transposed) tensor, the TT arithmetics referred to in the previous section, when applied to QTT decompositions, naturally provides the same basic operations in the QTT format.

Quantization is crucial for reducing the computational complexity further, as it allows the TT decomposition to resolve and represent more structure in the data by splitting the “virtual” dimensions introduced by the quantization, as well as the “physical” ones. In practice it appears the most efficient to use as fine a quantization (i.e. with small 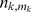) as possible and to generate as many virtual modes as possible. For example, when 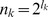 for 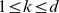, one may consider the “*ultimate quantization*” with 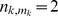 for all 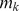 and 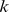. In this case, 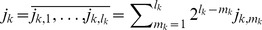, where the indices 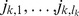 take the values 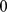 and 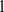.

The storage cost and complexity of basic QTT-structured operations are estimated from above through 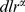 with 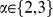, where 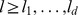 and 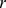 is an upper bound on all the QTT ranks of the decomposition in question. Note that this estimate may be, depending on 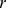, logarithmic in 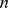 (also in 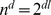, which is an upper bound on the number of entries). The notion of the effective rank defined by (10) for TT decompositions applies verbatim to vectors and matrices represented in the QTT format.

#### Structure of the CME operator in the QTT format

In the following we consider the Finite State Projection of the CME, as described previously, with 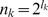 for 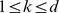 and assume that the PDF 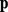 of the truncated model and of the CME operator 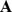 from (3) are represented in the QTT format outlined in the previous section. We use the ultimate quantization, so that 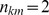 for 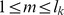 and 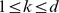. In this section we mathematically establish rigorous upper bounds on the QTT ranks of 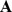 under certain assumptions on the propensity vectors 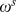, 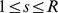, defined by (6).

**Theorem 4** *Consider the projected CME operator* 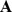 *defined by (3). Assume that for every* 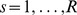 *and* 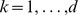 *the one-dimensional vector* 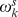 *from* (6)–(7) *is given in a QTT decomposition of ranks bounded by* 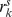 *; and that* 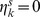 *implies* 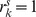 *. Then the CME operator* 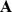 *admits a QTT decomposition of ranks*

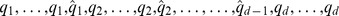

*with* 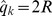 *for* 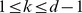 *and*

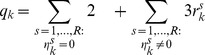

*for* 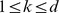.

*The proof is provided at the end of Text S1.*

A crude upper bound on the QTT ranks of the CME operator, following from Theorem 4 in terms of 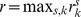, equals 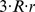 and is still favorable, since it ensures the estimate 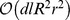 for the number of parameters, i.e. the storage cost, where 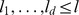. Note that if the 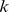
th factor 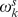 of the 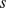-th propensity function is a polynomial of degree 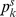, then 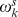 (7) can be represented in the QTT format with ranks bounded by 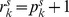 uniformly in 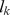, see [45, Corollary 13] and [42, Theorem 6]. In particular, this is the case when the reaction network is composed entirely of elementary reactions. Our numerical experiments show that the QTT ranks of propensity vectors corresponding to rational propensity functions are low as well, which results in low QTT ranks of the CME operator (in particular, see the toggle switch example).

The rank estimate of Theorem 4 is based on the construction of the CME operator, in which the reactions are treated independently, and the ranks of the terms corresponding to different reactions are summed. However, the bases of the QTT representation of these terms can be related so that the resulting decomposition of the CME operator can be reduced without introducing any error; for example, in the case of polynomial propensity functions. However, the rank bound of Theorem 4 is sharp for general vectors used as propensity vectors.

#### Transposed QTT representation

So far we have shown that the CME operator (3) under the FSP projection admits the low-parametric representation in the standard QTT format introduced previously. However, such a compressibility of the operator does not imply that the format is suitable for the efficient numerical solution of the CME. The example presented in Section S1.2 hints at a natural modification of the QTT decomposition. We represent in the TT format the quantized vector with virtual dimensions permuted so that the “virtual” indices corresponding to the same levels of quantization of different physical dimensions are adjacent; for example, for 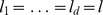 instead of (11) we use the ordering
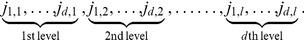
(12)When 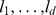 are not equal, in order to obtain a similar to (12) transposed ordering of indices, we introduce void indices 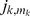 with 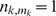 for 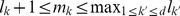, reorder all the “virtual” indices according to (12) and then drop the void ones. This modification of the QTT format, which we refer here to as *quantized-and-transposed Tensor Train*; shortly, *transposed QTT* or *QT3*. It was first applied to vectors in [55].

The index ordering (12) aims at the low-rank representation of such tensors, in which the physical dimensions are coupled on the corresponding virtual levels, i.e. *scales*, much more than different scales are within each single dimension. This is the case for the extreme example (S1.5), where we end up with a rank-one decomposition if we choose to separate the scales first, and the physical dimensions, then. Despite such a difference in approximation properties, from the algorithmic point of view, QT3 is a minor modification of the standard, widely used form of the QTT format. We do not imply any particular ordering of indices by referring simply to QTT.

#### Structure of the CME operator in the transposed QTT format

Similarly to Theorem 4, we can bound the ranks of the CME operator in the transposed QTT format relying on the ordering (12) of “virtual” indices.

**Theorem 5** *Consider the projected CME operator* 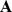 *defined by (3). Assume that for every* 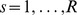 *and* 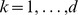 *the one-dimensional vector* 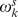 *from *(6)–(7)* is given in a QTT decomposition of ranks bounded by* 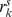 *; and that* 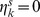 *implies* 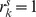 *. Then the CME operator* 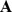 *admits a QT3 decomposition of ranks bounded by*

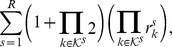

*where* 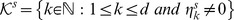.

*The proof is given at the end of Text S1.*

We observe in the enzymatic futile cycle example below that the QT3 ranks of the CME operator may be significantly lower than the bound of Theorem 4.

### Time Integration of the CME: hp-Discontinuous Galerkin Discretization

Let us consider the truncated CME (S1.1) with a state space 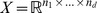 on a finite interval 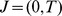. The Cauchy problem with an initial value 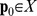 reads as find a continuously differentiable function 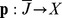 such that
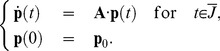
(13)The solution to (13) is given theoretically by 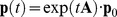 for 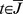, but the straightforward numerical evaluation of the matrix exponential involved is a very challenging task due to the “curse of dimensionality”. Instead, we use the QTT-structured 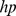-*discontinuous Galerkin* (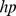 *-DG-QTT* for short) time-stepping scheme, proposed in [38], to solve (13). The 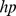-DG time stepping was proposed earlier in [56] for initial value problems for abstract, possibly non-linear, ODEs. We recapitulate the analysis results from [56] for problems of the particular form (13), which have unique, analytic in time classical solutions. To discuss the tensor structure of the 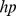-DG-QTT approach, we revisit [38].

Let us denote by 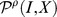 the space of polynomials defined on a finite interval 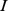, of degree 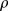 at most and with coefficients from 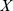. Let 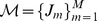 be a partition of the time interval 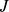 into subintervals 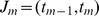, 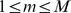, and 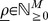. Consider the space
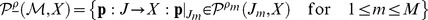
of functions, which are polynomials of degree 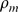 at most on 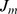 for all 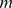. For all 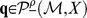 let 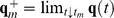 and 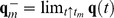 for all feasible 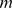.

#### Definition 6

*The* 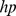 *-DG formulation of (13), corresponding to the partition* 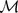 *of the time interval and the vector* 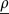 *of polynomial degrees, reads as follows: find* 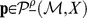 *such that*

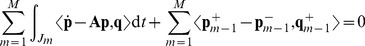
(14)
*for all* 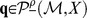 *, where* 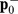 *stands for the initial value* 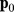.

Equation (14) can be understood as a time-stepping method: if for all 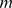 from 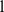 up to 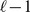 the polynomial 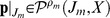 is known through 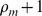 coefficients from 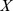, then 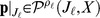 can be found as the solution to
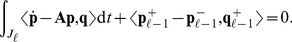
(15)For 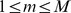 let 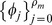 be a basis in 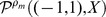, then the corresponding temporal shape functions on 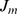 are 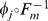, 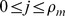, where the affine map 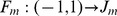 is defined by 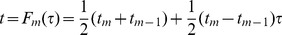 for 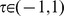. If 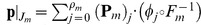, where 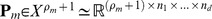, then (15) yields the following linear system on the coefficients:
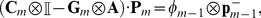
(16)where 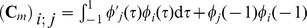 and 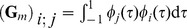 for 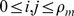, while 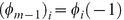 for 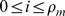.

The 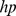-DG time discretization allows, on the one hand, to resolve fast transients in the evolution by the time-step and polynomial order adaptation for time-analytic solutions given through matrix exponentials of the CME operator. In particular, due to the time-analyticity of the solution, exponential rates of convergence in 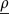 are achieved; for example, for the “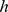-version” with 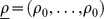 the error bound of Proposition 3 of Text S1 can be recast as
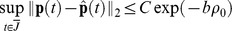
with constants 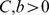 asymptotically independent of 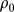, see [56, Theorem 3.18]. This implies that a prescribed level of accuracy 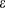 can be reached with 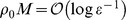 temporal degrees of freedom.

In the tensor representation of the system (16) we keep the QTT format used for 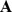 and attach the temporal index as a single dimension (without quantization) to the first “virtual” spatial index. In Section S1.3 we present this format in more detail.

#### Theorem 7

*Assume that* 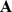 *is represented in the QTT or QT3 format in terms of* 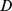 *cores with ranks* 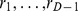 *. Then the matrix of system *(16)* can be represented in the corresponding format in terms of* 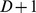 *cores with ranks* 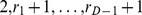.

*The proof is given at the end of Text S1.*

As an alternative to the presently considered order and stepsize adaptive time-stepping, it has been proposed in [5] to use a low-order time discretization with a uniformly small step and rely on tensor-structured compression methods also for time-adaptivity. This approach leads to one large linear system with low-rank structure. We found this approach to be more demanding to the tensor-structured solvers, since the aggregate linear system for all time steps seems to be more difficult to solve. A remedy may be to partition the time interval into subintervals with possibly different time steps being used within each such subinterval, which already shifts the approach in the direction of the presently proposed 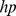-DG method. In the presence of time inhomogeneity the aggregate systems in general lose their low-rank structure rendering the space-time tensor approach less efficient, while the 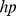-DG method would still perform well.

### Algorithm Summary

Assuming we have a finite state projection of the CME, we summarize our approach to the CME solution by outlining the two main algorithms we propose for its subsequent efficient solution. Given a reaction network and a finite state projection Algorithm 1 (Box 1) approximates the CME operator in QTT format. Algorithm 2 (Box 2) then describes the time-stepping procedure for computing the solution. Note that the integrals in Algorithm 2 may be pre-computed depending on the choice of temporal basis functions. E.g. if one chooses the Legendre polynomials as the basis, then there are explicit solutions of the integrals involved.

### Comparison to Krylov Subspace Methods

The solution at a particular time of a finite state projection of the CME is given analytically by the matrix exponential, but the numerical computation of such solutions for large 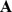 is often expensive. When 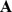 is sparse, however, the Krylov subspace method [57], [58] is one approach for performing the computation for the CME as described in [59]. The method uses the Arnoldi iteration to compute the Krylov subspace up to some order of accuracy then computes the matrix exponential in that smaller space (by diagonal Padé approximation). The publicly available Expokit Toolbox by Sidje [60] provides an implementation of the algorithm.

It is important to note that the algorithm steps incrementally in time rather than jumping to the desired time step. In the context of the CME, this means that the faster the support of the pdf fills the set of reachable states, the more expensive this algorithm becomes to compute. When there is reason to believe the support of the pdf remains small, then the algorithm can be expected to compute efficiently over large time intervals. Generically, however,the support of the pdf quickly fills the set of reachable states which may include every state retained in the projection. This renders the Arnoldi iteration computationally expensive at each time step.

The QTT method effectively circumvents this problem by storing the computed solution at each time step in the QTT format and exploiting the fast algorithms for basic tensor arithmetic available in this format. While it is unknown whether a given reaction network and initial probability distribution will produce an evolution that can be represented well by a QTT formatted tensor with low QTT ranks, our numerical experiments find this often is the case and that the savings over using traditional sparse representations of vectors and matrices may be quite substantial.

Below we compare our method to the Krylov subspace approach in the toggle switch example which does not exhibit any pronounced structure favoring either one of the methods (rank-one separability and sparse structure respectively).

### Numerical experiments

#### Common details

At the 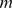
th time step, after having obtained 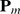 as an approximate solution of the corresponding linear system (16), we evaluate 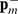 and reapproximate it in the TT format with relative 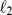-accuracy 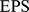 in order to drop excessive QTT components. The values of 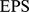 and the complete set of parameters of the time discretization and of the DMRG solver are reported for each experiment in Section S1.7.

We compare the evaluated solution or its marginal to a reference data. By 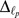 we denote the 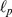-norm of the discrepancy. Generally we start with the 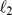-norm, which can be easily computed even when the comparison is made only in the (Q)TT format and cannot be made in the full format (which is the case in the 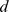-independent birth-death processes experiments for 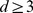). In some cases we compute also the discrepancy for 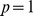 and the probability deficiency 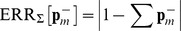. The reference data is also obtained with a certain accuracy which cannot be reduced arbitrarily. Moreover, in some cases our solution appears to be more accurate, which accounts for using the term “discrepancy” instead of “error”.

In the first and third examples we reapproximate the solution once more, with relative 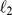-accuracy 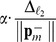, where 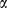 is 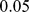 and 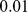 respectively. Below we refer to this procedure as *truncation*, and the approximated vector, as *truncated solution*. The procedure ensures that the relative discrepancy in the 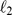-norm grows by the factor of 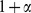 at most and shows what QTT ranks allow for our numerical solution, obtained without using any reference data, to ensure *almost* the same discrepancy from the reference data (which is related to the accuracy of both the solution and reference data) as before truncation.

#### *d* independent birth-death processes

As a first example we consider a system composed of 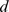 chemical species with 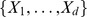 a vector of random variables representing the species count of each. The dynamics of the random vector are governed by independent birth-death processes. For the 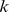-th species, the corresponding reactions are given by
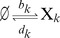
where 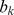 is the spontaneous creation rate and 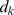 is the destruction rate for species 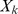. This problem is perfectly separable in the sense that the dynamics of any one chemical species of this system is independent of the dynamics of all others. Given the initial condition 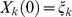 for each 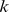, the marginal distribution for any one species 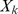 at time 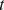 is given by:
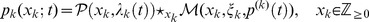
where 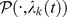 is the Poisson distribution with parameter 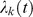, 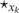 indicates the discrete convolution in variable 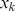, 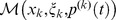 the multinomial distribution with parameter 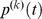, and the parameters 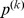 and 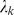 evolve according to the reaction rate equations
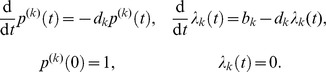
See [4, Theorem 1] for details. Since 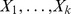 are mutually independent, the joint PDF at time 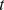, 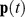, is the product of the marginals:
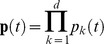
that is, this system has an explicit formula for the solution regardless of the number of chemical species involved. We can, therefore, evaluate the accuracy and observe the complexity scaling of the 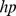-DG-QTT solver as the number of chemical species increases.

For numerical simulations we assume 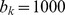 and 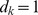 for 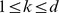 and consider the FSP with 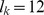. We solve the corresponding projected CME for 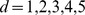 to check that in all these cases the 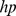-DG-QTT method using the ordering (11) without transposition is capable of revealing the same low-rank QTT structure of the solution. For the CME operator we have 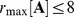 up to accuracy 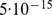. We compute the evolution of the PDF of the system for the zero initial value through 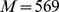 time steps till 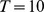.

**Figure 2:**
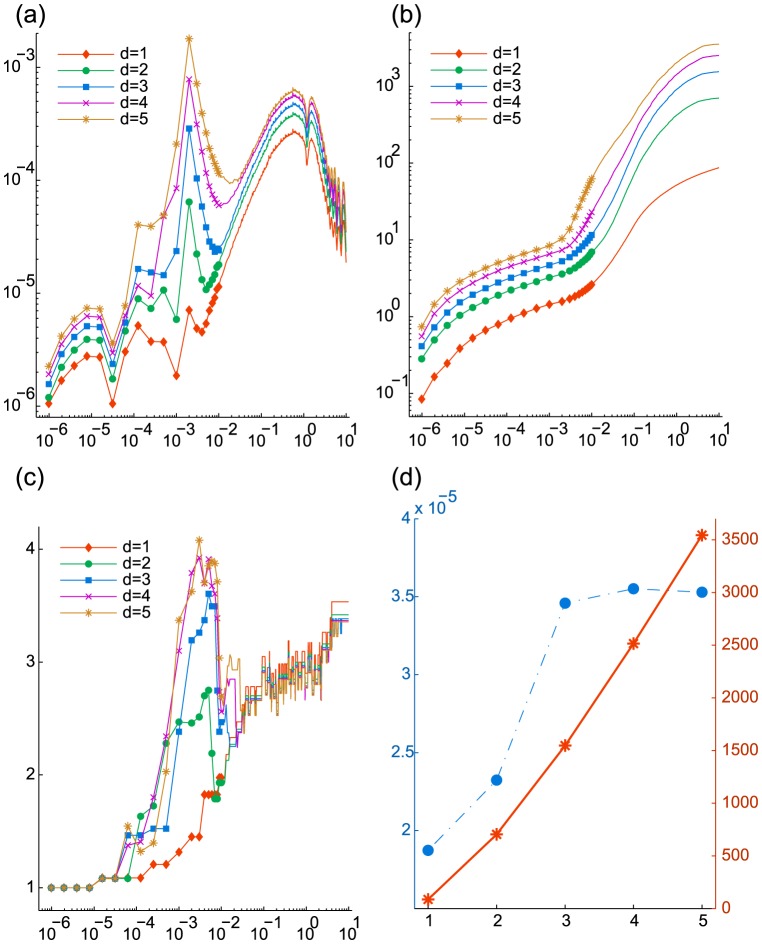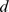 independent birth-death processes. The maximum QTT ranks of the solutions, 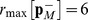 for each 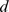. Markers are omitted for 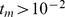 in (a)–(c). (a) Relative discrepancy 
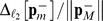
 (after truncation) vs. 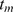. (b) Cumulative computation time (in seconds) vs. 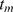. (c) Effective QTT rank 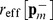 (after truncation) vs. 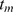. (d) Relative discrepancy 
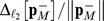
 (blue) and total computation time (red) vs. 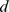.

The results, which are presented in Figure 2 and Table 2, show that the same low-rank structure of the solution is adaptively reconstructed by the algorithm for all 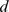 considered. The transient phase causes the growth of QTT ranks, because at certain steps of every sweep the DMRG solver merges virtual dimensions corresponding to different species and attempts to reduce the numerical rank by re-separating these dimensions. As a consequence, during the transient phase numerical QTT ranks are overestimated, which does not affect the QTT structure of the numerical solution at larger times.

**Table 2:** 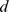
 independent birth-death processes: 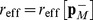, 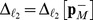, computational 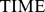 in seconds; 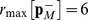 for all 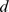.

*d*	*N*	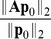	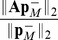	*r*_eff_	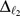	TIME
1	2^12^	1.4_+**3**_	1.0_−**3**_	3.53	1.9_−**5**_	87
2	2^24^	2.4_+**3**_	1.4_−**3**_	3.42	2.3_−**5**_	704
3	2^36^	3.5_+**3**_	1.8_−**3**_	3.38	3.5_−**5**_	1548
4	2^48^	4.5_+**3**_	2.0_−**3**_	3.37	3.6_−**5**_	2516
5	2^60^	5.5_+**3**_	2.3_−**3**_	3.36	3.5_−**5**_	3544

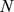
 is the number of states taken into account in the FSP. The exponents are given in boldface for the base 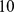.

#### Toggle switch

**Figure 3:**
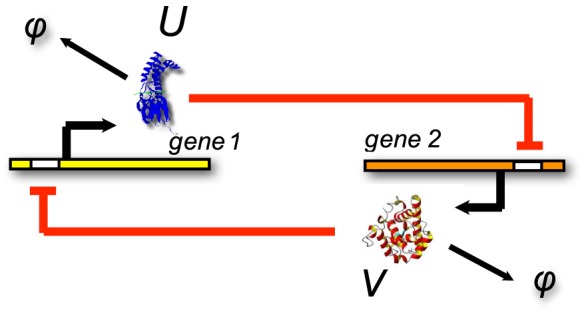
Toggle Switch consisting of double negative feedback loop. Species 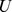 represses the production of species 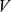 and vice versa.

The next example models a synthetic gene-regulatory circuit designed to produce bistability over a wide range of parameter values [61]. The network consists of two promoters constructed in a mutually inhibitory configuration that implement a double negative feedback loop, causing the network to exhibit robust bistable behavior (see Figure 3). If the concentration of one repressor is high, this lowers the production rate of the other repressor, keeping its concentration low. This allows a high rate of production of the original repressor, thereby stabilizing its high concentration.

A stochastic model of the toggle switch was considered in [62] and consists of the following four reactions:
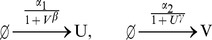


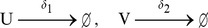
where 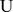 and 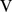 represent the two repressors. Denote the species counts of each by 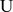 and 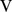, respectively. The stochastic model admits a bimodal stationary distribution over a wide range of values of the rate constants. We consider the set of parameters from [62] which were selected to test the efficiency of using available numerical algorithms to calculate matrix exponentials to solve low dimensional FSP approximations of the CME. We then scaled the parameters so that a larger set of states would be required to guarantee an FSP truncation with low approximation error. While a different set of parameters were considered in [23], [63], which required a larger FSP truncation, this choice of values renders the system symmetric under interchange of the roles of 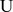 and 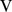. This situation is less biologically relevant than what we consider here.

For this numerical example we assume 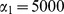, 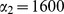, 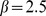, 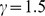, 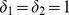. We consider the FSP with 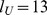, 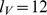, which allows to take into account 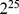 states. The initial value is zero. We use the ordering (11) without transposition. For the CME operator we have 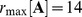 and 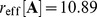 up to accuracy 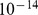. We compute the evolution of the PDF up to time 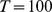 with 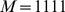 time steps.

**Figure 4:**
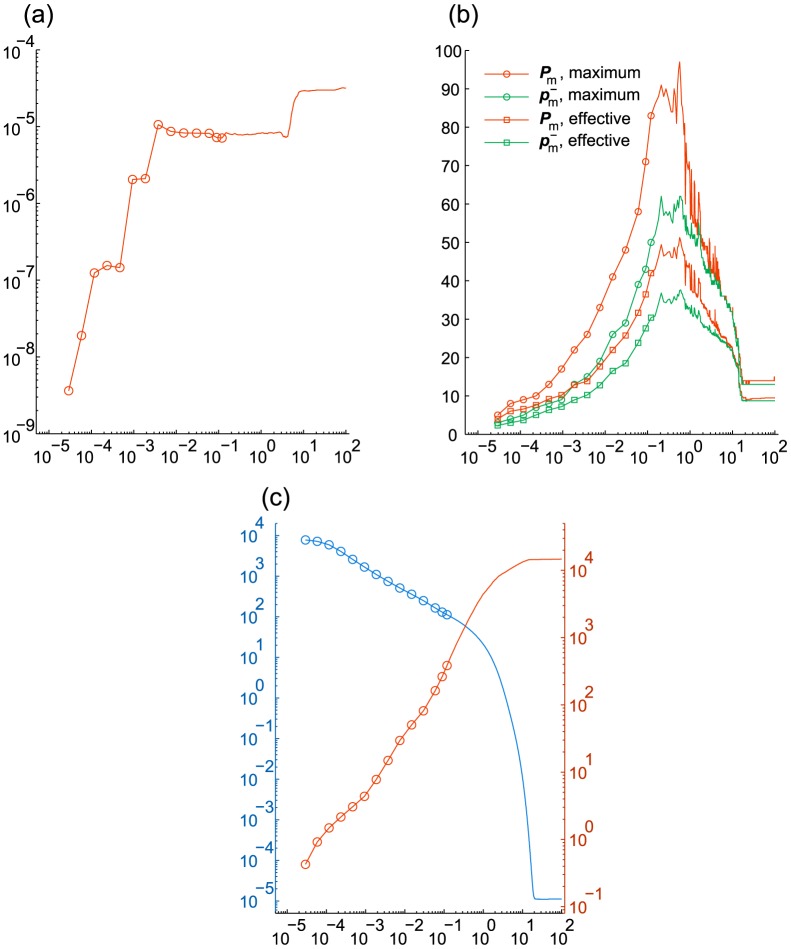
Genetic toggle switch. The values are given vs. 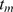. Markers are omitted for 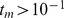. (a) Probability deficiency 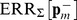. (b) Maximum and effective QTT ranks of the computed solution. (c) Relative norm 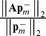 of the derivative (blue) and cumulative computation time (red, sec.)

**Figure 5:**
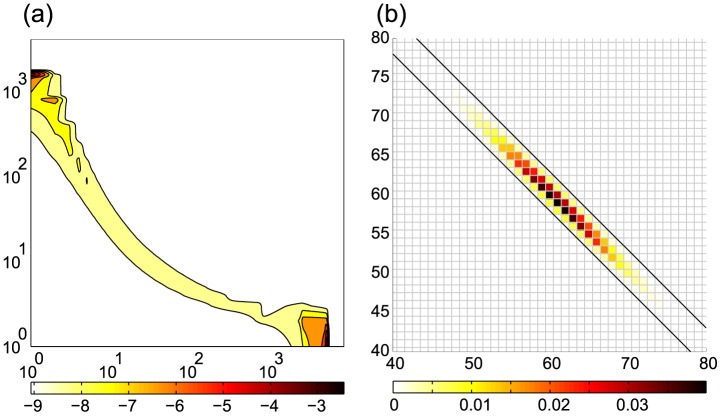
Snapshots of solutions. (a) Genetic toggle switch. The PDF for 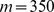, 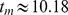, 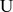 (hor.) vs. 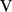 (vert.). As the process evolves, the probability mass becomes concentrated in two distinct regions. Contour coloring is logarithmically scaled with base 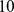. (b) Enzymatic futile cycle. The marginal PDF for 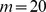, 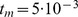, 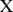 (vert.) vs. 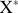 (hor.). Black diagonal lines delimit the states reachable from the initial condition. The transposed QTT format automatically exploits this sparsity pattern *of the full PDF* for compression without special input from the user.

The results are presented in Figure 4. At the terminal time 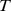 we have 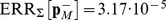. The overall computation time is 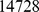 seconds. The validation with the PDF based on 816 million Monte Carlo simulations (every 1000 draws taking on average over 360 seconds, adding up to the overall CPU time over 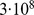 seconds), indicates 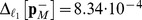, and for the 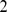
- and Chebyshev norms we have 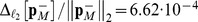 and 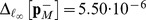. As for the ranks, 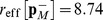 and 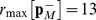. Figure 4 (c) shows that after 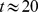 the norm of the time derivative stagnates at approximately 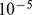 determined by the accuracy parameters chosen, and the following time steps require negligible computational effort. At the same time, as we see in Figure 4 (b), all QTT ranks stabilize under 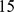, but the transient phase preceding that moment involves far higher ranks. Figure 5 (a) presents a snapshot of the distribution.

#### Comparison to the Krylov subspace approach

We compared the performance of our proposed method to that of the Krylov subspace approach implemented in Sidje's Expokit [60]. In order to make the comparison as fair as possible we further restricted the FSP truncation used by the Krylov approach to a *hyperbolic cross*, that is, we only kept states with indices 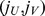 satisfying the condition 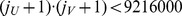. Effectively, this reduces the states in the truncation from 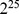 to 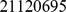, a reduction of about a third. A similar truncation was used for this model in [62].

We emphasize that formulating this hyperbolic cross truncation requires special insight into the problem on the part of the modeler. In constrast, our proposed method is completely naive in this respect, instead relying on the adaptivity of the QTT compression.

For the FSP with 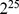 states considered we reach 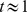 with the first 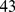 time steps of our method in 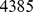 seconds; with the Krylov subspace method restricted to the hyperbolic cross, in 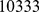 seconds. For the discrepancy between the two solutions obtained we have 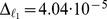 and 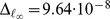.

At approximately 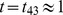, the decay of the relative norm of the solution becomes exponential; see Figure 4 (c). That is exploited by our method in two ways. On the one hand, we adjust the time mesh manually, which reduces the overall number of time steps needed to reach 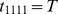 from 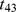: we take 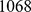 steps intead of approximately 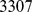 we would need if we had used a uniform time mesh for the long-term dynamics. On the other hand, what is more significant, the adaptive QTT representation used at each step yields a substantial speedup of the solution of linear systems, which is possible due to the rapid convergence of the solution to a stationary distribution. The Krylov subspace solver adapts the time mesh on its own, but employs no self-adaptivity for efficient storage of numerically computed states. As a result, the performance (in terms of the computational time vs. physical time of the system) decays much slower for the Krylov subspace solver, and our method excels even more in modelling the long-term dynamics. For example, our method achieves 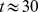, when 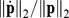 reaches 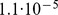, with the overall computation time 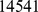 seconds compared to 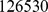 seconds of the Krylov subspace solver, i.e. approximately 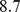 times faster. For larger terminal times the advantage of our method becomes even more pronounced.

#### Enzymatic futile cycle

**Figure 6:**
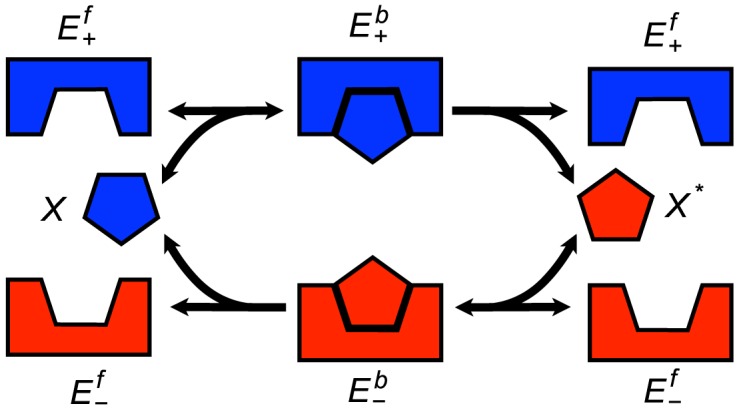
Enzymatic futile cycle. 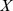
 is transformed into 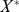 and vice versa by enzymes 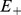 and 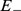, respectively.

Futile cycles are composed of two metabolic or signaling pathways that work in opposite directions so that the products of one pathway are the precursors of the other and vice versa, see Figure 6. This biochemical network structure results in no net production of molecules and often results only in the dissipation of energy as heat [64]. Nevertheless, there is an abundance of known pathways that use this motif and it is thought to provide a highly tunable control mechanism with potentially high sensitivity [64], [65].

[65] introduced a stochastic version of the model with just the essential network components required to model the dynamics. The stochastic model consists of six chemical species and six reactions:
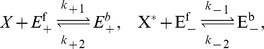


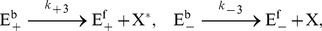


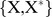
 represent the forward substrate and product, 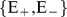 denote the forward and reverse enzymes, respectively. Note that this system is closed meaning that particles are neither created nor destroyed. We denote the random variables representing the molecule count of each species with italics.

For the particular set of initial conditions considered in [65] the number of states that are reachable is large enough to render a direct numerical solution of the CME impractical. The authors instead used the Gillespie Direct SSA to generate a large number of sample paths to estimate the distribution. The authors also applied a diffusion approximation to their model which resulted in a SDE which produced qualitatively similar dynamics. To the authors' knowledge, no attempt has been made so far towards the direct numerical solution of the CME for this system.

At time 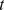, let 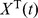 denote the total amount of both free and bound substrate, and 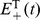 and 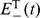 the total forward and reverse enzymes, respectively. We observe the following conservation relations:
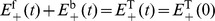


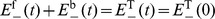


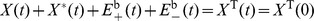
Using the above, one can establish an upper and lower bound relating the species count of 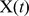 to 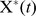 that depends only on the total initial amount of substrate and the total initial amount of enzymes in the system
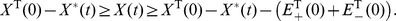
Assuming that the initial quantity of enzymes 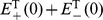 is small, for a given copy number of 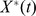, 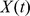 may take at most 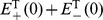 different values. Since 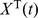 is a conserved quantity, this means that 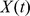 and 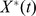 will be strongly anti-correlated with the set of reachable states having an affine structure. Under these circumstances, we find in our numerical experiments that the transposed QTT format is better suited than the standard QTT to efficiently represent the corresponding PDF, since the transposed format better utilizes the sparsity pattern of the full PDF for compression.

Following [65], we consider 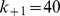, 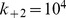, 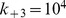, 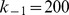, 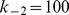, 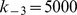. For initial value we take 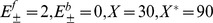. We consider the FSP projection with 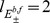 and 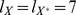, i.e. with 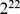 states. We present 4 runs: (A), (B) and (C) use the transposed QTT format, and (D), the standard QTT. Theorems 5 and 4 bound the exact QTT ranks of the CME operator by 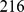 and 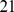 respectively, and numerically for accuracy 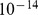 we have 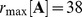, 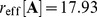 in (A)–(C) and 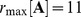, 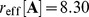 in (D). We compute the evolution of the PDF up to time 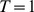 with 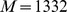 time steps.

For the runs (A) and (D), which differ in the format, we keep the same accuracy parameters. The runs (B) and (C) use the same format as (A), but different accuracy parameters, so that they yield, respectively, a more accurate and a cruder solution as compared to (A).

This experiment shows, in particular, that lower ranks of the operator do not necessarily lead to lower ranks of the solution, and that in this example the transposed QTT format actually ensures smaller ranks of the solution than the QTT format without transposition does and than Theorem 5 suggests. As for the solution, we observe that 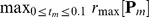 reaches 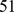 for (A) and 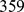 for (D).

**Figure 7:**
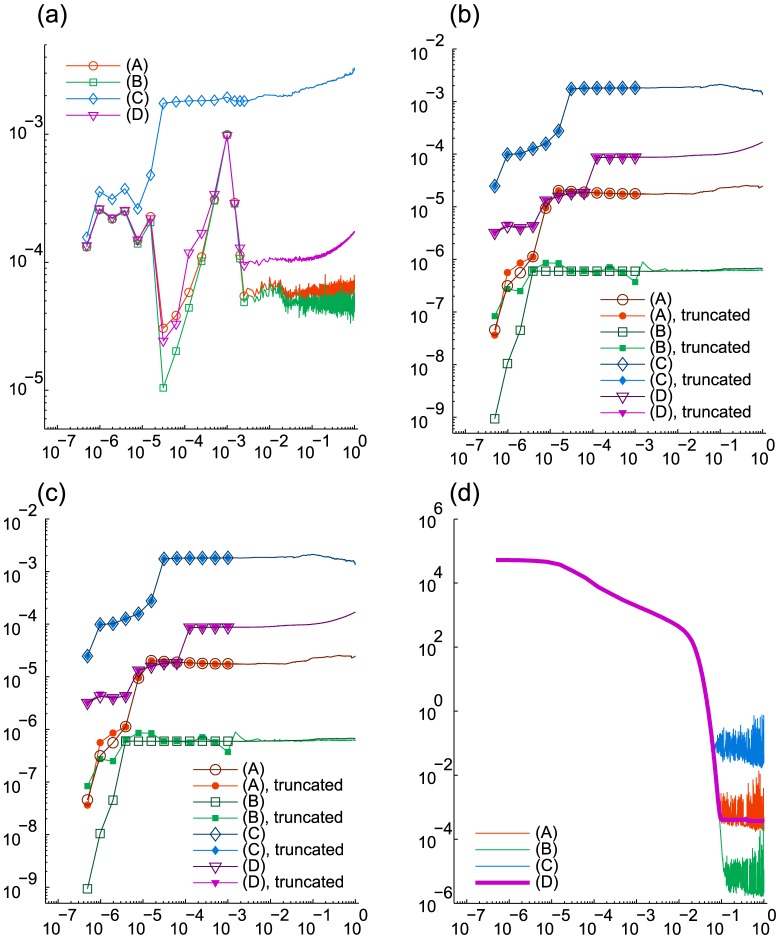
Enzymatic futile cycle. The values are given vs. 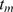. Markers are omitted for 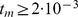 in (a)–(c). (a) Discrepancy 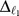 (before truncation) from the marginal PDF based on Monte Carlo simulations. (b) Probability deficiency 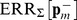. (c) Cumulative computation time (sec.) (d) Relative norm 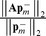 of the derivative.

For every 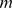, we validate our solution 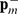 by comparing its marginal distribution 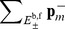 to that based on 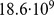 Monte Carlo simulations (every 10000 draws taking at least 110 seconds, amounting to the overall CPU time over 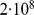 seconds). The discrepancy 
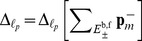
 in the marginal distribution with respect to 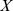 and 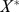 is reported for 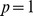 in Figure 7 (a) and Table 3. With 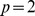 we use it for the discrepancy-based truncation, which, as Figure 7 (b) shows, does not affect the probability deficiency significantly.

**Table 3:** Enzymatic futile cycle: 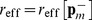, 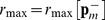, 
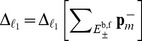
, 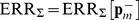 are given for the truncated solution 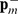; computational 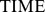 is given in seconds; 
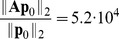
.

run	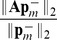	*r*_eff_	*r*_max_	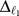	ERR_Σ_	TIME
*m* = 210, *t_m_* = 0.1						
(A)	3.5_−**4**_	13.17	27	5.7_−**4**_	2.3_−**5**_	1.07_3_
(B)	6.5_−**5**_	12.14	25	4.6_−**5**_	6.1_−**7**_	1.60**_3_**
(C)	1.3_−**1**_	12.16	24	2.3_−**3**_	2.1_−**3**_	9.87**_2_**
(D)	4.1_−**4**_	60.06	109	1.1_−**4**_	1.0_−**4**_	9.23**_3_**
*m* = *M* = 1322, *t_m_* = *T* = 1						
(A)	1.8_−**4**_	13.66	27	7.2_−**5**_	2.5_−**5**_	3.70**_3_**
(B)	1.1_−**5**_	12.06	25	5.7_−**5**_	6.2_−**7**_	4.21**_3_**
(C)	2.5_−**2**_	12.85	24	3.3_−**3**_	1.3_−**3**_	4.03**_3_**
(D)	3.7_−**4**_	58.97	107	1.7_−**4**_	1.7_−**4**_	1.52**_4_**

The exponents are given in boldface for the base 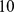.

Figure 7 (a) shows that the refined run (B) yields the smallest discrepancy, which suggests that the reference distribution is sufficiently accurate to allow for the discrepancy to represent the actual error in the results of (A), (B) and (C). As we can see from Figure 7 (d), in all 4 runs the time derivative stagnates after 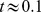, at lower levels for more accurate runs. Let us note that at that stage in (A)–(C) it exhibits relatively strong oscillations compared to (D), which happens due to different effect of the addition of random components in the DMRG solver in the presence and absence of the transposition. On the other hand, compared to (A), the run (D) yields a less accurate solution and reaches 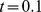 almost 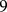 times later, the accuracy settings being the same in these two runs. In all, the transposition appears to make the QTT format far more efficient in this experiment, and we expect it to be even more so in larger systems of such type.

**Figure 8:**
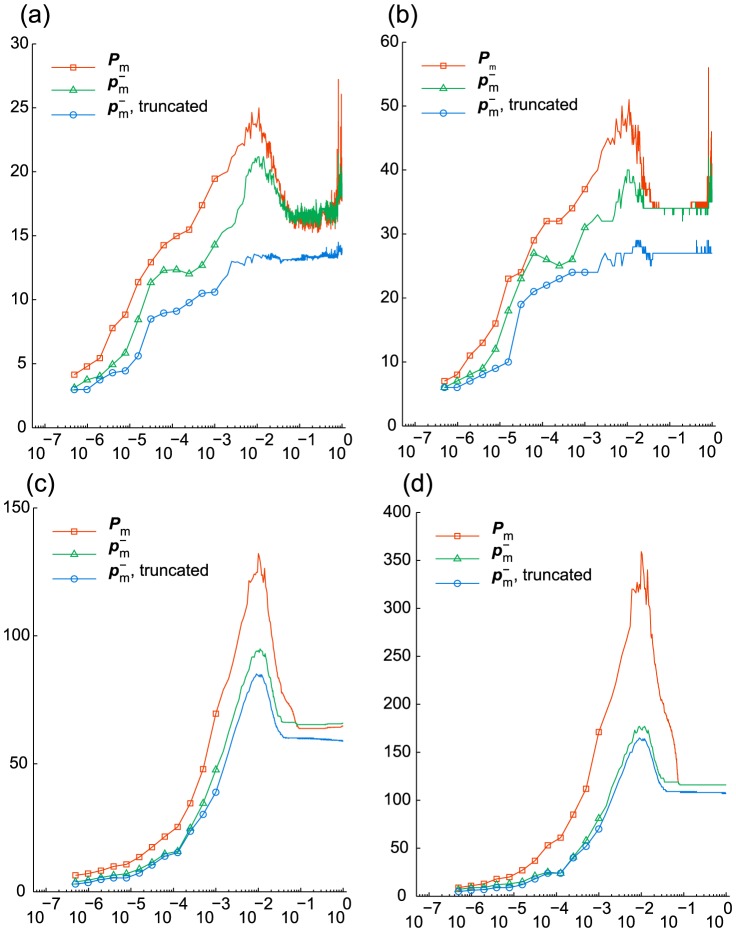
Enzymatic futile cycle. QTT ranks of the solution. The values are given vs. 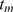. Markers are omitted for 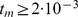. (a) Effective QTT ranks 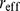 for parameter set (A). (b) Maximum QTT ranks 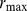 for parameter set (A). (c) Effective QTT ranks 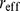 for parameter set (D). (d) Maximum QTT ranks 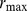 for parameter set (D).

The results are given in Figures 7 and 8 and in Table 3. Figure 5 (b) presents a snapshot of the marginal distribution.

### Conclusion

We presented a novel, “ab-initio” computational methodology for the direct numerical solution of the CME. The methodology exploits the time-analytic nature of solutions to the CME and the low-rank, tensor structure of the CME operator by combining an 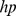-timestepping method that is order and step size adaptive, unconditionally stable and exponentially convergent with respect to the number of time discretization parameters, with novel, tensor-formatted linear algebra techniques for the numerical realization of the method. In particular, after an initial projection on a (sufficiently rich) finite state, the QTT representation allows for the dynamic adaptation of the effective state-space size, as well as of the principal components, or basis elements of the numerical representation of solution vectors in the numerical simulation of the time evolution of the CME solution. We emphasize that, while the performance of our approach is better when the solution can be approximated in the QTT format with a high degree of separability of the “physical” and “virtual” variables (i.e. with low TT ranks), the approach does not require a particular degree of separability, but instead reveals possibly present low TT rank in the solution at runtime. In the course of rank adaptation, the singular vectors, in the span of which the solution is approximated, are also adapted. Hence, the presently proposed approach is superior to fixed basis approaches (even when used with adaptivity), such as those reported in [19], [22], [23], [66]. The precise class of chemical reaction networks that lead to low TT rank in the solution tensor is currently unknown. To the extent that this rank increase during runtime, the effectiveness of the compression will be decreased, which could prove limiting for some problems. However, in this case other methods will be equally challenged. Identifying the architecture of the chemical reaction networks that lead to very low ranks is currently a research problem under investigation.

While the discussion following Theorem 4 relates to the case when the factors of the propensity functions are monomial, the approach presented herein applies equally well to models with propensity functions that are merely smooth enough. For example [67], gives bounds on the QTT ranks of the propensity functions and CME operator in the case of the stochastic mass-action and Michaelis–Menten kinetics with separable propensity functions. Also, the same work proves the bounds on the QTT ranks of product-form stationary distributions [68] of *weakly-reversible* reaction networks of *zero deficiency in the sense of Feinberg* [69]. Those bounds explain some of the experimental observations made in the present paper. Furthermore, the approach proposed is suitable for non-separable propensity functions. However, in that case the characterization of the rank structure of the CME operator needs to rely on some extra assumptions ensuring moderate QTT ranks, even though more general than separability, and Algorithm 1 needs to be altered accordingly.

The performance of the approach proposed essentially relies on the efficiency of the numerical solution of TT-structured linear systems of equations. In particular, a globally (or “less strictly locally”) convergent iterative solver would allow us to take larger time steps and to exploit the exponential convergence of the 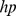-DG time discretization. We believe that while the presently reported numerical results which were obtained with the DMRG solver are quite encouraging, ongoing research on TT-structured linear system solvers holds the promise for a substantial efficiency increase of the present methodology. We only mention a family of alternating minimal energy methods which was announced very recently in [70].

We also mention that, of course, the choice of the tensor format and, possibly, index ordering, has an essential impact on the performance of the approach. The computational experiments reported in the present paper show that even a straightforward permutation of “virtual” indices produced by quantization may allow to exploit additional structure in the data and the QTT formatted CME solution and, therefore, may improve the performance of the QTT-structured approach dramatically. We point out that the TT format can be considered as a special case of *tensor network states*: TT formatted tensors belong to the class of simple, rooted tree-type tensor networks. Relating the architecture of the chemical reaction networks and appropriate tensor networks representing its states efficiently, i.e. with low ranks, is currently a research problem under development. The results of [67] mentioned above can be considered as the first step in this direction.

A general discussion of tensor networks and their use in numerical simulations for quantum spin systems can be found in [71], [72]. As for the numerical solution of the CME, particular real-life problems might require more sophisticated tensor networks to be used to efficiently approximate reachable states of the systems in question. The mathematical investigation of the relative merits and drawbacks of tensor formats for particular applications is currently undergoing rather active development; we mention only the recent monograph [40] and the references there.

We finally mention that recently, and independently, TT formatted linear algebra methods for the CME were proposed in [73]; a low order time stepping, and no transposition of tensor trains was used in that work. The CME examples presented in [73] also included a toggle switch, but the authors mostly rely on the intrinsic convergence of their method without analyzing actual accuracies. The latter are reported only for moderate sized examples which are computationally tractable with the direct approach in the full format. However, no attempt is made to analyze the accuracy in comparison to other simulation methods, which are typically applied to larger problems featuring essential difficulties for the direct approach. In the present paper we give comparisons with a state-of-the-art, massively parallel stochastic simulation package. This allows us, on the one hand, to validate the accuracy of the QTT-based solutions obtained here and, on the other hand, to provide evidence of the dramatic increase in efficiency afforded by the new deterministic approach: Monte Carlo simulations on 1500 cores of a high-performance cluster were matched in accuracy and outperformed in the wall-clock time by a MATLAB implementation running on a notebook.

## Methods

To solve the initial value problem for (2), we exploit the 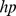-DG-QTT algorithm proposed in [38] and adapted to the CME as described above, implemented in MATLAB. It uses an implicit, exponentially convergent spectral time discretization of discontinuous Galerkin type. The resulting, time-discrete CME in “species space” is solved in the QTT format. Our implementation relies on the public domain *TT Toolbox* which provides basic TT-structured operations and solvers for linear systems in the QTT format. The TT toolbox is publicly available at http://spring.inm.ras.ru/osel and http://github.com/oseledets/TT-Toolbox; to be consistent, we use the GitHub version of July 12, 2012 in all examples below. We run the 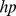-DG-QTT solver in MATLAB 7.12.0.635 (R2011a) on a laptop with a 2.7 GHz dual-core processor and 4 GB RAM, and report the computational time in seconds.

For the solution of the large, linear systems in the QTT and QT3 formats in each time step, we use the optimization solver, based on the DMRG approach [46]–[48] and elaborated on in the context of the TT format in [74] and available as the function dmrg_solve3 of the TT Toolbox. While the “DMRG” solver still lacks a rigorous theoretical foundation, it proves to be highly efficient in many applications, including our experiments. In [75] a closely related *Alternating Least Squares (ALS)* approach was mathematically analyzed and shown to converge locally. More on the mathematical ideas behind the ALS and DMRG optimization in the TT format can be found in [76].

The “DMRG” solver, under certain restrictions on the time step, manages to find a parsimonious QTT formatted solution of the linear system (up to a specified tolerance). Moreover, the solver in effect automatically adapts both the QTT rank as well as the QTT “basis” of the solution at every time step guaranteeing that it is sufficiently rich in order to capture the principal dynamics of interest.

In the first numerical example the solution is symmetric and exactly rank-one separable, which allows us to use the standard MATLAB solver ode15 s in the sparse format to obtain the univariate factor of a reference solution. In other examples we used SPSens beta 3.4, a massively parallel package for the stochastic simulation of chemical networks (http://sourceforge.net/projects/spsens/) [77], to construct reference PDFs. The stochastic simulations were carried out on up to 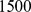 cores of Brutus, a high-performance cluster of ETH Zürich (https://www1.ethz.ch/id/services/list/comp_zentral/cluster/index_EN).

## Supporting Information

Text S1 Supplementary Material for direct solution of the Chemical Master Equations using Quantized Tensor Trains.
(PDF)
