# Supplementary material for: Direct Solution of the Chemical Master Equation Using Quantized Tensor Trains
Source: PLoS Comput Biol. 2014 Mar 13;10(3):e1003359. doi: 10.1371/journal.pcbi.1003359 (PMC3953644; doi:10.1371/journal.pcbi.1003359)
Supplement: Text S1 — Supplementary Material for direct solution of the Chemical Master Equations using Quantized Tensor Trains. (PDF) [file pcbi.1003359.s001.pdf]

# Direct Solution of the Chemical Master Equation using Quantized Tensor Trains[1]

Vladimir Kazeev[1], Mustafa Khammash[2*], Michael Nip[3], Christoph Schwab[4]
**1 Vladimir Kazeev, Seminar für Angewandte Mathematik, ETH Zürich. Rämistrasse 101, 8092 Zrich, Switzerland.**
**2 Mustafa Khammash, Department of Biosystems Science and Engineering, ETH Zürich. Mattenstrasse 26, 4058 Basel, Switzerland.**
**3 Michael Nip, Department of Mechanical Engineering, UC Santa Barbara. Engineering Building II, Santa Barbara, CA 93106-5070, USA**
**4 Christoph Schwab, Seminar für Angewandte Mathematik, ETH Zürich. Rämistrasse 101, 8092 Zrich, Switzerland.**
**∗ E-mail: mustafa.khammash@bsse.ethz.ch**

# Text S1

First, in Section S1.1, we recapitulate the result of [1] on the accuracy of the FSP approximation of the CME and, in Section S1.2, we provide some additional insight in the transposed QTT format. Then, in Section S1.3, we supplement the presentation of the *hp*-DG-QTT approach, given in the manuscript, with the analysis of the time discretization, based on [2], and with the discussion of the tensor structure of the linear systems resulting from the discretization. In Section S1.4 we revisit the notions of *core matrices* and *strong Kronecker product* according to the papers [3–5]. We use the notation introduced there to present in Section S1.5 some basic operations in the TT format. Finally, in Section S1.6 we provide proofs of Theorems 4, 5 and 7 and, therefore, for all theoretical assertions on QTT ranks made in the present paper. We note that the Theorems 4 and 5 are new mathematical results, and Lemma 10, on which the latter is based, may have applications well beyond the numerical solution of the CME.

## S1.1 Accuracy of the Finite State Projection

**Theorem 1** (Finite State Projection, Theorem 2.2 in [1])**.** *Consider a Markov process with state space* $\mathbb{Z}^d_{\geq 0}$ *whose probability density evolves according to the initial value ODE: given an initial state* $\mathbf{p}_0 \in [0,1]^{\mathbb{Z}^d_{\geq 0}}$*, find* $\mathbf{p}(t) \in [0,1]^{\mathbb{Z}^d_{\geq 0}}$ *such that*

$$\frac{\mathrm{d}}{\mathrm{d}t}\,\mathbf{p}(t) = \mathbf{A}\mathbf{p}(t) \quad 0 \leq t < \infty\ , \qquad \mathbf{p}(0) = \mathbf{p}_0$$

*where the operator* $\mathbf{A} : [0,1]^{\mathbb{Z}^d_{\geq 0}} \mapsto [0,1]^{\mathbb{Z}^d_{\geq 0}}$ *can be interpreted as a semi-infinite matrix.*

*Let* $\mathbf{A}^{\underline{n}}$ *denote the restriction of* $\mathbf{A}$ *to* $\Omega^{\underline{n}}$ *defined by* (5) *and assume that* $\mathbf{p}_0$ *is supported in* $\Omega^{\underline{n}}$*, i.e. that* $\mathbf{p}_0 = 0$ *in* $\mathbb{Z}^d_{\geq 0}\backslash\Omega^{\underline{n}}$*. Denote by* $\hat{\mathbf{p}}.(\cdot) \in [0,1]^{\Omega^{\underline{n}}}$ *the solution of the truncated system with dynamics given by the linear ODE:*

$$\frac{\mathrm{d}}{\mathrm{d}t}\,\hat{\mathbf{p}}(t) = \mathbf{A}^{\underline{n}}\hat{\mathbf{p}}(t)\ , \qquad 0 \leq t < \infty \tag{S1.1}$$

*with initial condition* $\hat{\mathbf{p}}_{\underline{x}}(0) = \mathbf{p}_{\underline{x}}(0) = \mathbf{p}_0(\underline{x})$*. If for some* $\epsilon > 0$ *and* $\tau \geq 0$

$$\sum_{\underline{x}\in\Omega^{\underline{n}}} \hat{\mathbf{p}}_{\underline{x}}(\tau) \geq 1 - \epsilon \tag{S1.2}$$

[1]Partially supported by the European Research Council (ERC) FP7 programme project AdG247277, NSF Grant ECCS-0835847, and the Human Frontier Science Program Grant RGP0061/2011. The funders had no role in study design, data collection and analysis, decision to publish, or preparation of the manuscript.

*then*

$$\hat{\mathbf{p}}_{\underline{x}}(\tau) \leq \mathbf{p}_{\underline{x}}(\tau) \leq \hat{\mathbf{p}}_{\underline{x}}(\tau) + \epsilon \tag{S1.3}$$

*for every* $\underline{x} \in \Omega^{\underline{n}}$ *and*

$$\left\| \hat{\mathbf{p}}(\tau) - \mathbf{p}(\tau)\,|_{\Omega^{\underline{n}}} \right\|_1 \leq \epsilon. \tag{S1.4}$$

Assume that a truncation satisfying (S1.2) can be found, then (S1.4) gives an explicit certificate of the accuracy of the approximate solution.

## S1.2 Transposed QTT representation

The following example demonstrates a simple example of non-axis-parallel features in the data, which cannot be represented in the format with low ranks. Our numerical experiments show that such features in the data may arise in systems with a conservation relationship between two or more chemical species resulting in a strong correlation in their copy numbers.

To illustrate this, let us consider the identity matrix and its vectorization:

$$\mathbf{A}_{i;\,j} = \mathbf{u}_{\overline{ij}} = \delta(i,j) \quad \text{for} \quad 1 \leq i,j \leq n, \tag{S1.5}$$

where $n = 2^l$. The matrix $\mathbf{A}$, which is the only TT unfolding and the $l$th QTT unfolding of the vector $\mathbf{u}$, is of full rank. This implies that an exact representation of $\mathbf{u}$ in the TT and QTT formats will have at least one rank equal to $2^l$ and cannot represent $\mathbf{u}$ efficiently. However, the matricization $\mathbf{A}$ is perfectly separable:

$$\mathbf{A} = \begin{pmatrix} 1 & & & \\ & 1 & & \\ & & \ddots & \\ & & & 1 \end{pmatrix} = \begin{pmatrix} 1 & 0 \\ 0 & 1 \end{pmatrix}^{\otimes l},$$

with a QTT matrix decomposition consisting of $l$ cores $V_k$ of size $1 \times 2 \times 2 \times 1$, given by

$$V_k(1,\cdot,\cdot,1) = \begin{pmatrix} 1 & 0 \\ 0 & 1 \end{pmatrix}$$

and with $l-1$ QTT ranks equal to $1,\ldots,1$. Further, we can also separate the $i$- and $j$-indices in each factor and end up with a representation with $2l$ factors and $2l-1$ ranks, all being bounded by 2.

In other words, the indices $i = \overline{i_1,\ldots,i_l}$ and $j = \overline{j_1,\ldots,j_l}$ may not be separable at all, while the mixed and re-ordered indices $\overline{i_1,j_1},\ldots,\overline{i_l,j_l}$ are perfectly separable. The ordering of the multi-index should reflect the structure in the data to achieve an optimal compression.

The transposed QTT representation was first applied to vectors in [6]; namely, to vectors of the form

$$\mathbf{u}_{j_1,\ldots,j_d} = \begin{cases} 1, & \sum_{k=1}^{d} j_k \leq 2^l, \\ 0, & \text{otherwise,} \end{cases}$$

where $\underline{j} = (j_1,\ldots,j_d) \in \left\{1,\ldots,2^l\right\}^d$. Such a vector may be considered as a discretization of the indicator function of the simplex $\left\{x \in \mathbb{R}^d_{\geq 0} : \|x\|_1 \leq 1\right\}$. In [6], $\mathbf{u}$ was shown to have a QT3 decomposition of ranks bounded linearly in $d$ uniformly in $l$. In the particular case of $d = 2$ such a bound follows from the result of [4] on the structure of Toeplitz matrices generated by tensor-structured vectors.

## S1.3 *hp*-DG-QTT discretization of the CME

Let us denote $|\mathcal{M}| = \max_{1\le m\le M}(t_m - t_{m-1})$. A fixed point argument (valid even for nonlinear evolution equations with Lipschitz nonlinearity) was used in [2] to prove the following result.

**Proposition 2** (Theorem 2.6 in [2])**.** *Assume that* $\|\mathbf{A}\|_2 \cdot |\mathcal{M}| < 1$. *Then there exists a unique solution to the linear equations* (14) *which results from the hp-DG time discretization of the CME.*

This existence result was complemented in [2] by a convergence rate estimate for the DG solutions.

**Proposition 3.** *Let* $\hat{\mathbf{p}}$ *and* $\mathbf{p}$ *be solutions of* (13) *and* (14) *respectively. Then*

$$\sup_{t\in \bar{J}} \|\mathbf{p}(t) - \hat{\mathbf{p}}(t)\|_2 \le C(\|\mathbf{A}\|_2, T)\cdot \widetilde{C}(\underline{\rho})\cdot \max_{1\le m\le M}\left[(c|\mathcal{M}|)^{\rho_m+1}\cdot \rho_m^{-\rho_m-\frac{1}{4}}\cdot \exp\rho_m\right]$$

*holds with a positive constant* $c>0$, *where* $|\underline{\rho}| = \max_{1\le m\le M}\rho_m$ *and* $\widetilde{C}(\underline{\rho}) = \log^{\frac{1}{2}}\max\{2, |\underline{\rho}|\}$.

*The proof follows from Theorem 3.12 in [2] in the analytic case, and from Stirling's formula.* □

Let us assume that the matrix $\mathbf{A}$ is represented in the QTT or QT3 format in terms of $\tilde{d}$ cores. In particular, if $n_k = 2^{l_k}$ for $1\le k\le d$, then $\tilde{d} = l_1 + \ldots + l_d$ for the ultimate quantization. The system (16) is of order $(\rho_m+1)\times n_1\times\ldots\times n_d$, where the first dimension indexes the components corresponding to the temporal shape functions on $J_m$. In the tensor representation of the system and its solution we keep the temporal index as a single dimension (without quantization) connected to the first "virtual" spatial index, so that $\mathbf{P}_m$ is indexed by the tuples

$$\underbrace{j,}_{\text{time dim.}}\ \underbrace{j_{1,1},\ldots,j_{1,l_1}}_{\text{1st dimension}},\underbrace{j_{2,1},\ldots,j_{2,l_2}}_{\text{2nd dimension}},\ldots\ldots,\underbrace{j_{d,1},\ldots,j_{d,l_d}}_{d\text{th dimension}} \tag{S1.6}$$

and

$$\underbrace{j,}_{\text{time dim.}}\ \underbrace{j_{1,1},\ldots,j_{d,1}}_{\text{1st level}},\underbrace{j_{1,2},\ldots,j_{d,2}}_{\text{2nd level}},\ldots\ldots,\underbrace{j_{1,l},\ldots,j_{d,l}}_{d\text{th level}} \tag{S1.7}$$

in the QTT and QT3 formats respectively, cf. (11) and (12). The right-hand side of (16) is formed by attaching $\phi_m$ to a QTT or QT3 decomposition of $\mathbf{p}_{-}^{m-1}$, therefore the first rank of the resulting decomposition is equal to 1 and the rest $\tilde{d}$ are the same as for $\mathbf{p}_{-}^{m-1}$. Theorem 7 shows that the matrix of (16) can be trivially represented in such a format with the first rank equal to 2 and each remaining rank equal to 1 plus the corresponding rank of $\mathbf{A}$. The proof is given at the end of this Supplementary material.

## S1.4 Core matrices and the strong Kronecker product

By a *TT core* of rank $r_{k-1}\times r_k$ and mode size $m_k\times n_k$ we denote an array of real numbers, which has size $r_{k-1}\times m_k\times n_k\times r_k$. The first and the last indices of a core are called (respectively, *left* and *right*) *rank indices*, while the others are referred to as *mode indices.* Subarrays of a core, corresponding to particular values of rank indices, have size $m_k\times n_k$ and are called *TT blocks.* We may consider the core $V_k$ as an $r_{k-1}\times r_k$-matrix with TT blocks as elements:

$$V_k = \begin{bmatrix} G_{11} & \cdots & G_{1r_k} \\ \vdots & \vdots & \vdots \\ G_{r_{k-1}1} & \cdots & G_{r_{k-1}r_k}\end{bmatrix} = \left[G_{\alpha_{k-1}\alpha_k}\right]_{\substack{\alpha_{k-1}=1,\ldots,r_{k-1}\\ \alpha_k=1,\ldots,r_k}}, \tag{S1.8}$$

where $G_{\alpha_{k-1}\alpha_k}$, $\alpha_{k-1} = 1, \dots, r_{k-1}$, $\alpha_k = 1, \dots, r_k$ are TT blocks of $V_k$, i.e. $V_k(\alpha_{k-1}, i_k, j_k, \alpha_k) = \left(G_{\alpha_{k-1}\alpha_k}\right)_{i_k j_k}$ for all values of rank indices $\alpha_{k-1}, \alpha_k$ and mode indices $i_k, j_k$. We refer to this matrix as *core matrix* of $V_k$.

In order to avoid confusion, we use parentheses for ordinary matrices, whose entries are numbers and which are multiplied as usual, and square brackets for cores (core matrices), whose entries are blocks and which are multiplied by means of the strong Kronecker product "$\bowtie$" defined below. Addition of cores is meant elementwise. Also, we may think of $G_{\alpha\beta}$ or of any submatrix of the core matrix in (S1.8) as *subcores* of $V_k$. For example, given matrices or cores $U_{11}, U_{12}, U_{21}, U_{22}$ of equal mode size and compatible ranks, we may use them as subcores to compose the cores

$$\begin{bmatrix} U_{11} & U_{12} \\ U_{21} & U_{22} \end{bmatrix} \quad \text{and} \quad \begin{bmatrix} U_{11} & \\ & U_{12} \end{bmatrix} = \operatorname{diag}\left[U_{11}, U_{22}\right].$$

We leave zero blocks blank, as in the last equation.

To ease notation, we omit in TT decompositions like (8), (9) the mode indices with the help of the *strong Kronecker product* [7]. To avoid the confusion with the Hadamard and tensor products, we denote this operation by "$\bowtie$", as in [3, Definition 2.1], where it was introduced as follows, specifically for connecting cores into "tensor trains".

**Definition 4** (Strong Kronecker product $\bowtie$ of TT cores)**.** *Consider cores $V_1$ and $V_2$ of ranks $r_0 \times r_1$ and $r_1 \times r_2$ and of mode sizes $m_1 \times n_1$ and $m_2 \times n_2$ respectively, composed of blocks $G^{(1)}_{\alpha_0\alpha_1}$ and $G^{(2)}_{\alpha_1\alpha_2}$, $1 \le \alpha_k \le r_k$ for $0 \le k \le 2$. Then the* strong Kronecker product $V_1 \bowtie V_2$ *of $V_1$ and $V_2$ is defined as core of rank $r_0 \times r_2$ and mode size $m_1 m_2 \times n_1 n_2$, consisting of blocks*

$$G_{\alpha_0\alpha_2} = \sum_{\alpha_1=1}^{r_1} G^{(1)}_{\alpha_0\alpha_1} \otimes G^{(2)}_{\alpha_1\alpha_2}, \quad 1 \le \alpha_0 \le r_0, \quad 1 \le \alpha_2 \le r_2.$$

In other words, we define $V_1 \bowtie V_2$ as a usual matrix product of the corresponding core matrices, their entries (blocks) being multiplied by means of the Kronecker (tensor) product. For example,

$$\begin{bmatrix} G_{11} & G_{12} \\ G_{21} & G_{22} \end{bmatrix} \bowtie \begin{bmatrix} H_{11} & H_{12} \\ H_{21} & H_{22} \end{bmatrix} = \begin{bmatrix} G_{11} \otimes H_{11} + G_{12} \otimes H_{21} & G_{11} \otimes B_{12} + G_{12} \otimes H_{22} \\ G_{21} \otimes H_{11} + G_{22} \otimes H_{21} & G_{21} \otimes B_{12} + G_{22} \otimes H_{22} \end{bmatrix}.$$

Equation (9) can be written then as

$$\mathbf{A} = V_1 \bowtie V_2 \bowtie \dots \bowtie V_{d-1} \bowtie V_d. \tag{S1.9}$$

In the particular case when the second mode length is 1 in each core, the strong Kronecker product of them is a vector and the second mode indices can be omitted. For example, equation (9) reads

$$\mathbf{p} = U_1 \bowtie U_2 \bowtie \dots \bowtie U_{d-1} \bowtie U_d. \tag{S1.10}$$

## S1.5 Some operations in the TT format

In this section we present a few basic operations in the TT format. The results given for matrices are valid for vectors. Vice versa, the statements formulated for vectors hold for matrices too. Indeed, the latter can be vectorized by merging their mode indices, subjected to the operations in question, and the result can be turned back into a matrix.

**Proposition 5** (Section 3.1 in [8])**.** *If a vector $\mathbf{p}$ is given in a CP decomposition*

$$\mathbf{p} = \sum_{\alpha=1}^{r} G_{1,\alpha} \otimes \dots \otimes G_{d,\alpha},$$

*it can be represented in the TT format as* $\mathbf{p} = U_1 \bowtie \ldots \bowtie U_d$ *with* $U_k = \operatorname{diag}[G_{k,1}, \ldots, G_{k,r}]$ *for* $2 \le k \le d-1$,

$$U_1 = \begin{bmatrix} G_{1,1} & \ldots & G_{1,r} \end{bmatrix} \quad \text{and} \quad U_d = \begin{bmatrix} G_{d,1} \\ \vdots \\ G_{d,r} \end{bmatrix}.$$

In particular, the TT ranks are bounded by the CP rank.

**Proposition 6** (Section 4.1 in [8])**.** *Assume that* $\mathbf{p} = U_1 \bowtie \ldots \bowtie U_d$ *and* $\mathbf{q} = V_1 \bowtie \ldots \bowtie V_d$ *are vectors of equal mode size, then a linear combination of them can be written as follows*

$$\alpha\mathbf{p} + \beta\mathbf{q} = \begin{bmatrix} U_1 & V_1 \end{bmatrix} \bowtie \operatorname{diag}[U_2, V_2] \bowtie \ldots \bowtie \operatorname{diag}[U_{d-1}, V_{d-1}] \bowtie \begin{bmatrix} \alpha U_d \\ \beta V_d \end{bmatrix}$$

*for all* $\alpha, \beta \in \mathbb{R}$.

Thus, the ranks of such a decomposition of $\alpha\mathbf{A} + \beta\mathbf{B}$ are sums of the corresponding ranks of the given decompositions of $\mathbf{A}$ and $\mathbf{B}$.

**Proposition 7.** *If a vector* $\omega$ *is given in the TT format through* $\omega = U_1 \bowtie \ldots \bowtie U_d$*, then its diagonalization* $\operatorname{diag}\omega$ *can be represented in the TT format as* $\operatorname{diag}\omega = V_1 \bowtie \ldots \bowtie V_d$*, where the cores of the matrix are obtained by diagonalizing all the blocks in every core of the vector:* $V_k(\alpha, i, j, \beta) = U_k(\alpha, j, \beta) \cdot \delta(i, j)$ *for all* $\alpha, i, j, \beta$ *and for* $1 \le k \le d$.

Therefore, TT ranks are preserved under diagonalization.

**Proposition 8** (Section 4.3 in [8])**.** *Consider matrices* $\mathbf{A}$ *and* $\mathbf{B}$ *given in TT representations* $\mathbf{A} = U_1 \bowtie \ldots \bowtie U_d$ *and* $\mathbf{B} = V_1 \bowtie \ldots \bowtie V_d$ *of ranks* $p_1, \ldots, p_{d-1}$ *and* $q_1, \ldots, q_{d-1}$ *respectively. Let* $p_0 = p_d = q_0 = q_d = 1$ *and assume that for* $1 \le k \le d$ *the cores* $U_k = \begin{bmatrix} A_{\alpha_{k-1}\alpha_k} \end{bmatrix}_{\substack{\alpha_{k-1}=1,\ldots,p_{k-1} \\ \alpha_k=1,\ldots,p_k}}$ *and* $V_k = \begin{bmatrix} B_{\beta_{k-1}\beta_k} \end{bmatrix}_{\substack{\beta_{k-1}=1,\ldots,q_{k-1} \\ \beta_k=1,\ldots,q_k}}$ *are of such mode size that all matrix-matrix products* $C_{\overline{\alpha_{k-1}\beta_{k-1}}\,\overline{\alpha_k\beta_k}} = A_{\alpha_{k-1}\alpha_k} \cdot B_{\beta_{k-1}\beta_k}$ *are correctly defined. Then the matrix-matrix product* $\mathbf{A} \cdot \mathbf{B}$ *has a TT decomposition* $\mathbf{A} \cdot \mathbf{B} = W_1 \bowtie \ldots \bowtie W_d$ *with*

$$W_k = \begin{bmatrix} C_{\overline{\alpha_{k-1}\beta_{k-1}}\,\overline{\alpha_k\beta_k}} \end{bmatrix}_{\substack{\alpha_{k-1}=1,\ldots,p_{k-1},\beta_{k-1}=1,\ldots,q_{k-1} \\ \alpha_k=1,\ldots,p_k,\beta_k=1,\ldots,q_k}}$$

*and ranks* $p_1q_1, \ldots, p_{d-1}q_{d-1}$.

*The proof.* The claim is obtained by writing the matrix-matrix product elementwise in terms of TT cores and in changing the summation order. □

For our considerations it is important that the corresponding TT ranks are multiplied under matrix-matrix multiplication.

**Proposition 9.** *Consider vectors* $\mathbf{p}$ *and* $\mathbf{q}$ *given in TT decompositions* $\mathbf{p} = U_1 \bowtie \ldots \bowtie U_d$ *and* $\mathbf{q} = V_1 \bowtie \ldots \bowtie V_d$*. The tensor product* $\mathbf{p} \otimes \mathbf{q}$ *can be written as* $\mathbf{p} \otimes \mathbf{q} = U_1 \bowtie \ldots \bowtie U_d \bowtie V_1 \bowtie \ldots \bowtie V_d$.

In particular, the ranks of the first factor are followed by the ranks of the second factor with 1 in between. In what follows, we denote the operation of tensor transposition which was described in Section , by $\mathcal{T}$.

**Lemma 10.** *Consider vectors* $\mathbf{p}$ *and* $\mathbf{q}$ *given in TT decompositions* $\mathbf{p} = U_1 \bowtie \ldots \bowtie U_d$ *and* $\mathbf{q} = V_1 \bowtie \ldots \bowtie V_d$ *of ranks* $p_1, \ldots, p_{d-1}$ *and* $q_1, \ldots, q_{d-1}$ *respectively. The transposed tensor product* $\mathcal{T}(\mathbf{p} \otimes \mathbf{q})$ *has a TT decomposition* $\mathcal{T}(\mathbf{p} \otimes \mathbf{q}) = \overline{U}_1 \bowtie \overline{V}_1 \bowtie \overline{U}_2 \bowtie \overline{V}_2 \bowtie \ldots \bowtie \overline{U}_{d-1} \bowtie \overline{V}_{d-1} \bowtie \overline{U}_d \bowtie \overline{V}_d$ *of ranks*

$$p_1, p_1q_1,\ p_2q_1, p_2q_2,\ \ldots,\ p_{d-2}q_{d-2}, p_{d-1}q_{d-2},\ p_{d-1}q_{d-1}, q_{d-1}$$

*with* $\overline{U}_1 = U_1$, $\overline{V}_d = V_d$ *and the other cores defined as follows:*

$$\begin{aligned}\overline{V}_1\big(\zeta_1, j_1, \overline{\eta_1\beta_1}\big) &= V_1(j_1,\beta_1)\cdot\delta(\zeta_1,\eta_1)\,,\\ \overline{U}_d(\overline{\alpha_{d-1}\mu_{d-1}}, i_d, \nu_{d-1}) &= U_d(\alpha_{d-1}, i_d)\cdot\delta(\mu_{d-1},\nu_{d-1})\end{aligned}$$

*and, for* $2 \le k \le d-1$,

$$\begin{aligned}\overline{U}_k(\overline{\alpha_{k-1}\mu_{k-1}}, i_k, \overline{\alpha_k\nu_{k-1}}) &= U_k(\alpha_{k-1}, i_k, \alpha_k)\cdot\delta(\mu_{k-1},\nu_{k-1})\,,\\ \overline{V}_k\big(\overline{\zeta_k\beta_{k-1}}, j_k, \overline{\eta_k\beta_k}\big) &= V_k(\alpha_{k-1}, j_k, \alpha_k)\cdot\delta(\zeta_k,\eta_k)\end{aligned}$$

*for all mode indices* $i_k, j_k$, *where* $1 \le k \le d$, *and for* $1 \le \alpha_k, \zeta_k, \eta_k \le p_k$ *and* $1 \le \beta_k, \mu_k, \nu_k \le q_k$, *where* $1 \le k \le d-1$.

*Proof.* By changing the order of summation and multiplication, for all values of mode indices $i_1, \dots, i_d$, $j_1, \dots, j_d$ we obtain

$$\begin{aligned}&(\mathbf{p}\otimes\mathbf{q})_{\,i_1,\dots,i_d,j_1,\dots,j_d}\\ &= \sum_{\alpha_1=1}^{p_1}\cdots\sum_{\alpha_{d-1}=1}^{p_{d-1}} U_1(i_1,\alpha_1)\cdot U_2(\alpha_1,i_2,\alpha_2)\cdot\ldots\\ &\quad\cdot U_{d-1}(\alpha_{d-2},i_{d-1},\alpha_{d-1})\cdot U_d(\alpha_{d-1},i_d)\\ &\quad\cdot\sum_{\beta_1=1}^{q_1}\cdots\sum_{\beta_{d-1}=1}^{q_{d-1}} V_1(j_1,\beta_1)\cdot V_2(\beta_1,j_2,\beta_2)\cdot\ldots\\ &\quad\cdot V_{d-1}(\beta_{d-2},j_{d-1},\beta_{d-1})\cdot V_d(\beta_{d-1},j_d)\\ &= \sum_{\alpha_1=1}^{p_1}\sum_{\beta_1=1}^{q_1}\cdots\sum_{\alpha_{d-1}=1}^{p_{d-1}}\sum_{\beta_{d-1}=1}^{q_{d-1}} U_1(i_1,\alpha_1)\cdot V_1(j_1,\beta_1)\\ &\quad\cdot U_2(\alpha_1,i_2,\alpha_2)\cdot V_2(\beta_1,j_2,\beta_2)\cdot\ldots\\ &\quad\cdot U_{d-1}(\alpha_{d-2},i_{d-1},\alpha_{d-1})\cdot V_{d-1}(\beta_{d-2},j_{d-1},\beta_{d-1})\cdot\\ &\quad\cdot U_d(\alpha_{d-1},i_d)\cdot V_d(\beta_{d-1},j_d)\\ &= (\mathcal{T}\,(\mathbf{p}\otimes\mathbf{q}))_{\,i_1,j_1,\dots,i_d,j_d}.\end{aligned}$$

□

**Lemma 11.** *Let* $U$ *be a core of rank* $p_0 \times p_d$ *with a* $d$*-dimensional mode index. For* $r \in \mathbb{N}$ *consider the core* $\overline{U}$ *defined by setting*

$$\overline{U}\big(\overline{\alpha_0\gamma_0}, \overline{i_1,\dots,i_d}, \overline{\alpha_d\gamma_d}\big) = U\big(\alpha_0, \overline{i_1,\dots,i_d}, \alpha_d\big)\cdot\delta(\gamma_0,\gamma_d)$$

*for all values of mode indices* $i_1, \dots, i_d$, *for* $1 \le \alpha_0 \le p_0$, $1 \le \alpha_d \le p_d$ *and for* $1 \le \gamma_0, \gamma_d \le r$.

*Assume that* $U$ *is given in a decomposition* $U = U_1 \bowtie U_2 \bowtie \ldots \bowtie U_{d-1} \bowtie U_d$, *where* $U_k$ *is of rank* $p_{k-1} \times p_k$. *Then* $\overline{U}$ *can be represented as* $\overline{U} = \overline{U}_1 \bowtie \overline{U}_2 \bowtie \ldots \bowtie \overline{U}_{d-1} \bowtie \overline{U}_d$, *where for* $1 \le k \le d$ *the core* $\overline{U}_k$ *of rank* $p_{k-1}r \times p_k r$ *is defined as follows:*

$$\overline{U}_k(\overline{\alpha_{k-1}\gamma_{k-1}}, i_k, \overline{\alpha_k\gamma_k}) = U_k(\alpha_{k-1}, i_k, \alpha_k)\cdot\delta(\gamma_{k-1},\gamma_k)$$

*for all values of mode index* $i_k$, *for* $1 \le \alpha_{k-1} \le p_{k-1}$, $1 \le \alpha_k \le p_k$ *and for* $1 \le \gamma_{k-1}, \gamma_k \le r$.

*Proof.* For all rank and mode indices we have

$$
\begin{aligned}
&\overline{U}\left(\overline{\alpha_0\gamma_0}, \overline{i_1, \ldots, i_d}, \overline{\alpha_d\gamma_d}\right) \\
&= \sum_{\alpha_1=1}^{p_1} \ldots \sum_{\alpha_{d-1}=1}^{p_{d-1}} \delta(\gamma_0, \gamma_d) \prod_{k=1}^{d} U_k(\alpha_{k-1}, i_k, \alpha_k) \\
&= \sum_{\gamma_1=1}^{r} \sum_{\alpha_1=1}^{p_1} \ldots \sum_{\gamma_{d-1}=1}^{r} \sum_{\alpha_{d-1}=1}^{p_{d-1}} \prod_{k=1}^{d} \delta(\gamma_{k-1}, \gamma_k)\, U_k(\alpha_{k-1}, i_k, \alpha_k)\,,
\end{aligned} \tag{S1.11}
$$

i.e. $\overline{U} = \overline{U}_1 \bowtie \overline{U}_2 \bowtie \ldots \bowtie \overline{U}_{d-1} \bowtie \overline{U}_d$. □

**Corollary 12.** *Assume that vectors* $\mathbf{p}_k$ *,* $1 \le k \le d$*, are given in QTT decompositions with* $l$ *quantization levels and of ranks* $r_1^{(k)}, \ldots, r_{l-1}^{(k)}$*,* $1 \le k \le d$*, respectively. Then the tensor product* $\mathbf{p}_1 \otimes \ldots \otimes \mathbf{p}_d$ *can be represented in the transposed QTT format with ranks*

$$
\begin{gathered}
r_1^{(1)},\ r_1^{(1)} r_1^{(2)},\ \ldots,\ r_1^{(1)} \cdot r_1^{(2)} \cdot \ldots \cdot r_1^{(d-1)} \cdot r_1^{(d)}, \\
{\color{blue} r_1^{(1)} r_1^{(2)} \cdot \ldots \cdot r_1^{(d-1)} \cdot r_1^{(d)}}, \\
r_2^{(1)} \cdot r_1^{(2)} \cdot \ldots \cdot r_1^{(d)} \cdot r_1^{(d)},\ \ldots,\ r_2^{(1)} \cdot r_2^{(2)} \cdot \ldots \cdot r_2^{(d)} \cdot r_1^{(d)}, \\
{\color{blue} r_2^{(1)} r_2^{(2)} \cdot \ldots \cdot r_2^{(d-1)} \cdot r_2^{(d)}}, \\
\ldots\ldots \\
{\color{blue} r_{l-2}^{(1)} r_{l-2}^{(2)} \cdot \ldots \cdot r_{l-2}^{(d-1)} \cdot r_{l-2}^{(d)}}, \\
r_{l-1}^{(1)} \cdot r_{l-2}^{(2)} \cdot \ldots \cdot r_{l-2}^{(d)} \cdot r_{l-2}^{(d)},\ \ldots,\ r_{l-1}^{(1)} \cdot r_{l-1}^{(2)} \cdot \ldots \cdot r_{l-1}^{(d-1)} \cdot r_{l-2}^{(d)}, \\
{\color{blue} r_{l-1}^{(1)} r_{l-1}^{(2)} \cdot \ldots \cdot r_{l-1}^{(d-1)} \cdot r_{l-1}^{(d)}}, \\
r_{l-1}^{(2)} \cdot \ldots \cdot r_{l-1}^{(d)},\ \ldots,\ r_{l-1}^{(d-1)} \cdot r_{l-1}^{(d)},\ r_{l-1}^{(d)},
\end{gathered}
$$

*where we highlight in blue the QT3 ranks separating adjacent levels. As a result, all QT3 ranks of the tensor product are bounded from above by*

$$
\prod_{k=1}^{d} \max_{1 \le m \le l-1} r_m^{(k)}.
$$

*The proof follows from Lemmas 10 and 11.* □

When the numbers of levels of quantization vary, i.e. $l_1 = \ldots = l_d = l$ does not hold for any $l$, Corollary 12 still remains true. Indeed, one can increase the number of cores in each decomposition up to $\max_{1 \le k \le d} l_k$ by introducing void cores with void mode indices and of rank $1 \times 1$ (so that these cores are just constant factors), apply the results presented above and remove the void cores by contracting them with the others.

## S1.6 Proofs of the theorems

*Proof of Theorem 4.* Assume $1 \le s \le R$. The corresponding shift matrix is a Kronecker product:

$$
\mathbf{S}_{\underline{\eta}^s} = \mathbf{S}_{\eta_1^s}^{(l_1)} \otimes \ldots \otimes \mathbf{S}_{\eta_d^s}^{(l_d)}\,,
$$

where $\mathbf{S}_{\eta_k^s}^{(l_k)}$ is the $2^{l_k}\times 2^{l_k}$-matrix of the zero-filling $\eta_k^s$-position shift, $1\le k\le d$. By [4, Lemma 4.2], each of these one-dimensional factors can be represented explicitly in the QTT format with ranks bounded by 2 for any $\eta_k^s$. However, if $\eta_k^s=0$, then $\mathbf{S}_{\eta_k^s}^{(l_k)}=\mathbb{I}$ is of QTT ranks $1,\ldots,1$. Therefore, according to Proposition 9, the QTT ranks of $\mathbf{S}_{\underline{\eta}^s}$ are bounded by $\rho_1^s,\ldots,\rho_1^s$, $1$,$\rho_2^s,\ldots,\rho_2^s$, $1,\ldots,\ldots,1$,$\rho_d^s,\ldots,\rho_d^s$, where

$$\rho_k^s=\begin{cases}2, & \eta_k^s\neq 0,\\ 1, & \eta_k^s=0\end{cases}$$

for $1\le k\le d$. As the identity matrix is of QTT ranks $1,\ldots,1$, by Proposition 6, the QTT ranks of $\mathbf{S}_{\underline{\eta}^s}-\mathbb{I}$ are bounded by $\rho_1^s+1,\ldots,\rho_1^s+1$, $2$,$\rho_2^s+1,\ldots,\rho_2^s+1$, $2,\ldots,\ldots,2$,$\rho_d^s+1,\ldots,\rho_d^s+1$.

Analogously we obtain that the QTT ranks of $\omega^s$ are bounded by $r_1^s,\ldots,r_1^s$, $1$,$r_2^s,\ldots,r_2^s$, $1,\ldots,\ldots,1$, $r_d^s,\ldots,r_d^s$, where for $1\le k\le d$ we have $r_k^s=1$ if $\eta_k^s=0$. Due to Proposition 7, the same bounds hold true for the QTT ranks of the matrix $\operatorname{diag}\omega^s$.

Finally, we use Proposition 8 to conclude that the $s$th term $\left(\mathbf{S}_{\underline{\eta}^s}-\mathbb{I}\right)\circ\mathbf{M}_{\omega^s}$ of the CME operator is represented in the QTT format with ranks bounded by $\tilde{q}_1^s,\ldots,\tilde{q}_1^s$, $2$,$\tilde{q}_2^s,\ldots,\tilde{q}_2^s$, $2,\ldots,\ldots,2$,$\tilde{q}_d^s,\ldots,\tilde{q}_d^s$, where

$$\tilde{q}_k^s=\begin{cases}3\cdot r_k^s, & \eta_k^s\neq 0,\\ 2\cdot 1, & \eta_k^s=0\end{cases}$$

for $1\le k\le d$. By summing these rank bounds, we obtain the rank bounds claimed for $\mathbf{A}$ with $q_k=\sum_{s=1}^R\tilde{q}_k^s$ and $\hat{q}_k=\sum_{s=1}^R 2$. □

*Proof of Theorem 5.* Analogous to that of Theorem 4. For the QT3 format, we use Corollary 12 instead of Proposition 9 to construct tensor products and to establish the corresponding rank bounds. □

*Proof of Theorem 7.* Let us set $U_0=[\mathbf{C}_m]$, $U_m=[\mathbb{I}_{\rho_m+1}]$ for $1\le m\le\sum_{k=1}l_k$, $V_0=[-\mathbf{G}_m]$ and assume $\mathbf{A}=U_1\bowtie\ldots\bowtie U_{\sum_{k=1}l_k}$. Then the proof follows from Proposition 6.

□

## S1.7 Parameters of the DMRG solver

In this section we present the parameters of the time discretization and of the DMRG solver, used in the numerical experiments.

The fact that the DMRG solver, as any other tensor-structured solver available, converges only locally, requires the time steps to be rather small to allow for the corresponding linear systems being solved. For this reason, we have to use an equidistant mesh of mesh width $h$ on $[h,T_1]$, where the transient processes are strong, but on $[T_1,T]$ can increase the mesh width geometrically with the grading factor $\sigma_2=\frac{t_{m-1}}{t_m}=1-\frac{h}{T_1}$, which is only slightly less than 1. On $[0,h]$ we initialize our algorithm by $M_0=10$ steps graded geometrically with the factor $\sigma_0=\frac{t_{m-1}}{t_m}=0.5$. On all time intervals we use polynomial spaces of degree $\rho=3$ to discretize (2) as described below, since the aforementioned limitation of the DMRG solver prevents us from using high polynomial degrees and enjoying the exponential convergence of the time discretization. For the bases in polynomial spaces corresponding to the time steps we take the orthonormal system of normalized Legendre polynomials.

In the numerical experiments, the following parameters of the DMRG solver are involved: the required relative residual `RES` of the linear system, the maximum number `SWP` of its iterations ("sweeps"), the maximum number `RST` of GMRES restarts for the "local problem" of the DMRG optimization procedure, the maximum number `ITR` of such iterations before a restart, the maximum feasible rank `RMX`, the rank `KCK` of random components added to the solution to avoid stagnation. The DMRG iterations continue until either their number reaches `SWP` or the relative residual is less than or equal to `RES`. In every particular run all those parameters are the same for all time steps.

### S1.7.1 $d$ Independent Birth-Death Processes

In this experiment $T = 10$, $T_1 = 10^{-1}$ and $h = 10^{-3}$. The overall number of time steps is $M = 569$.

The settings of the DMRG solver are: $\texttt{RES} = 2 \cdot 10^{-6}$, $\texttt{SWP} = 2$, $\texttt{RMX} = 20$, $\texttt{ITR} = 100$, $\texttt{RST} = 1$, $\texttt{KCK} = 1$. The evaluation accuracy is $\texttt{EPS} = 10^{-8}$.

### S1.7.2 Toggle Switch

In this experiment $T = 100$, $T_1 = 10$ and $h = 0.03$. The overall number of time steps is $M = 1111$.

The settings of the DMRG solver are: $\texttt{RES} = 10^{-6}$, $\texttt{SWP} = 3$, $\texttt{RMX} = 200$, $\texttt{ITR} = 100$, $\texttt{RST} = 2$, $\texttt{KCK} = 2$. The evaluation accuracy is $\texttt{EPS} = 10^{-8}$.

### S1.7.3 Enzymatic Futile Cycle

In this experiment $T = 1$, $T_1 = 0.3$ and $h = 5 \cdot 10^{-4}$. The overall number of time steps is $M = 1332$.

For (A) and (D), which differ in the format, we keep the same accuracy parameters: $\texttt{RES} = 10^{-6}$ and $\texttt{EPS} = 10^{-8}$. On the other hand, (B) and (C) use the same format as (A), but different accuracy parameters. In (B) they are $\texttt{RES} = 10^{-8}$ and $\texttt{EPS} = 10^{-10}$; in (C), $\texttt{RES} = 10^{-4}$ and $\texttt{EPS} = 10^{-6}$. We set $\texttt{RMX} = 200$ in (A)–(C) and $\texttt{RMX} = 400$ in (D). Other parameters of the DMRG solver are the same for all 4 runs: $\texttt{SWP} = 5$, $\texttt{ITR} = 50$, $\texttt{RST} = 2$, $\texttt{KCK} = 2$.

## References


1. Munsky B, Khammash M (2006) The finite state projection algorithm for the solution of the chemical master equation. The Journal of Chemical Physics 124: 044104.

2. Schötzau D, Schwab C (2000) An hp a priori error analysis of the DG time-stepping method for initial value problems. Calcolo 37: 207–232.

3. Kazeev VA, Khoromskij BN (2012) Low-rank explicit QTT representation of the Laplace operator and its inverse. SIAM Journal on Matrix Analysis and Applications 33: 742–758.

4. Kazeev VA, Khoromskij BN, Tyrtyshnikov EE (2011) Multiulevel Toeplitz matrices generated by tensor-structured vectors and convolution with logarithmic complexity. Preprint 36, Max-Planck-Institut für Mathematik in den Naturwissenschaften. URL `http://www.mis.mpg.de/publications/preprints/2011/prepr2011-36.html`.

5. Kazeev V, Reichmann O, Schwab C (2013) Low-rank tensor structure of linear diffusion operators in the TT and QTT formats. Linear Algebra and its Applications .

6. Oseledets IV (2010) QTT decomposition of the characteristic function of a simplex. Personal communication.

7. Launey WD, Seberry J (1994) The strong kronecker product. Journal of Combinatorial Theory, Series A 66: 192–213.

8. Oseledets IV (2011) Tensor Train decomposition. SIAM Journal on Scientific Computing 33: 2295–2317.
